# Pan-immune-inflammation value for risk stratification of adverse cardiovascular outcomes in acute coronary syndrome undergoing percutaneous coronary intervention: a systematic review and meta-analysis

**DOI:** 10.3389/fimmu.2026.1870678

**Published:** 2026-06-01

**Authors:** Xinrui Yin, Shijia Du

**Affiliations:** 1 Department of Anesthesiology, Aerospace Center Hospital, Beijing, China; 2 Department of VIP Dental Service, Peking University Stomatological Hospital, Beijing, China

**Keywords:** acute coronary syndrome, inflammation biomarker, major adverse cardiovascular events, meta-analysis, pan-immune-inflammation value, percutaneous coronary intervention, prognosis, systematic review

## Abstract

**Background:**

Patients with acute coronary syndrome (ACS) undergoing percutaneous coronary intervention (PCI) remain at residual cardiovascular risk. The pan-immune-inflammation value (PIV), integrating neutrophil, platelet, monocyte, and lymphocyte counts, has been proposed as a candidate prognostic biomarker in this population, but the evidence has not been formally synthesised. We evaluated the prognostic and discriminatory value of PIV in adults with ACS undergoing PCI.

**Methods:**

PubMed/MEDLINE, Embase, Web of Science Core Collection, the Cochrane Library, Scopus, and CNKI were searched from inception, supplemented by trial registries, Google Scholar, and reference hand-searching. Cohort studies reporting associations between PIV and major adverse cardiovascular events (MACE; primary outcome), all-cause mortality, cardiovascular or cardiac mortality, angiographic no-reflow or slow-flow, or post-contrast renal injury were eligible. Two reviewers independently performed study selection, data extraction, and QUIPS risk-of-bias assessment. Adjusted hazard ratios, odds ratios, and AUC values were pooled separately using random-effects models; certainty was rated with GRADE. PROSPERO: CRD420261378751.

**Results:**

Nineteen cohort studies (n = 18,715) were included. Higher PIV was associated with study-defined MACE (adjusted HR 1.65, 95% CI 1.20–2.27; k = 2; I² = 0.0%; low certainty) and all-cause mortality (HR 3.51, 95% CI 2.15–5.74; k = 2; I² = 0.0%; moderate certainty). A single study reported an association with cardiac mortality (HR 3.24, 95% CI 1.34–7.81; low certainty). PIV showed consistent discrimination for no-reflow or slow-flow (AUC 0.828, 95% CI 0.808–0.846; k = 2; I² = 0.0%; moderate certainty) and heterogeneous discrimination for post-contrast renal injury (AUC 0.771, 95% CI 0.617–0.875; k = 5; I² = 95.1%; very low certainty). Risk of bias was moderate in 16 studies and high in three.

**Conclusion:**

Elevated PIV may be associated with adverse cardiovascular outcomes in ACS patients undergoing PCI, particularly long-term all-cause mortality and angiographic no-reflow or slow-flow. However, evidence is limited by observational designs, small numbers of studies for several outcomes, heterogeneous cut-offs, and risk-of-bias concerns. PIV should be regarded as a promising supplementary marker requiring prospective multicentre validation, comparison with established inflammatory indices, and assessment of incremental value beyond GRACE or TIMI before routine clinical use.

**Systematic Review Registration:**

https://www.crd.york.ac.uk/prospero/, identifier CRD420261378751

## Introduction

Acute coronary syndrome (ACS), encompassing ST-elevation myocardial infarction (STEMI), non-ST-elevation myocardial infarction (NSTEMI), and unstable angina, remains a leading cause of cardiovascular mortality and morbidity worldwide (1). Percutaneous coronary intervention (PCI) is central to contemporary reperfusion and invasive management strategies for ACS and has substantially improved short-term survival (2). Nonetheless, even with timely revascularisation and contemporary antithrombotic and secondary prevention regimens, ACS patients undergoing PCI continue to experience residual risk of adverse outcomes. Composite major adverse cardiovascular events (MACE), all-cause and cardiovascular mortality, angiographic no-reflow or slow-flow during the index procedure, and post-contrast renal injury represent clinically important sources of early and long-term risk after PCI (3). Identifying patients at higher risk at or before the index procedure remains an important clinical priority that could inform risk stratification, in-hospital monitoring intensity, and post-discharge surveillance.

The pathophysiology underlying these adverse outcomes is increasingly understood as an integrated immune-thromboinflammatory process rather than a purely thrombotic or ischaemic event (4, 5). Neutrophils, platelets, monocytes, and lymphocytes participate in interrelated rather than parallel pathways spanning innate immune activation, thromboinflammation, vascular inflammation, post-infarction remodelling, and adaptive immune regulation, which have been implicated in microvascular obstruction, post-contrast renal injury, and longer-term cardiovascular adverse events (6, 7).

Against this background, peripheral blood-derived inflammatory indices have been proposed as accessible candidate biomarkers for risk stratification in ACS patients undergoing PCI. The neutrophil-to-lymphocyte ratio (NLR), platelet-to-lymphocyte ratio (PLR), systemic immune-inflammation index (SII; platelet × neutrophil/lymphocyte), and systemic inflammation response index (SIRI; neutrophil × monocyte/lymphocyte) have each been linked to adverse outcomes in this population (8–10). The pan-immune-inflammation value (PIV), originally introduced in oncology and subsequently adopted in cardiovascular research, integrates four cellular components—neutrophils, platelets, monocytes, and lymphocytes—within a single composite expression: PIV = (neutrophil × platelet × monocyte)/lymphocyte (11). This four-component structure differs from earlier indices, which integrate two (NLR, PLR) or three (SII, SIRI) cellular populations. A growing body of primary studies has examined PIV in ACS patients undergoing PCI (12–18), reporting associations of variable magnitude and robustness, while direct head-to-head comparisons with related inflammatory indices and assessments of incremental value beyond established clinical risk scores have been limited.

Although several studies have evaluated PIV in ACS patients undergoing PCI, their findings remain difficult to interpret together because of differences in study design, outcome definitions, follow-up duration, PIV measurement timing, and cut-off values. A systematic review and meta-analysis is therefore needed to clarify the prognostic and discriminatory value of PIV in this population using methods that separate effect-measure types, apply prognostic-factor-specific risk-of-bias and certainty assessment, and integrate both adjusted prognostic association estimates and discriminatory performance estimates across outcomes. We therefore conducted a prospectively registered systematic review and meta-analysis to evaluate the prognostic and discriminatory value of PIV for adverse cardiovascular outcomes in adult ACS patients undergoing PCI. The primary objective was to quantify the association between elevated PIV and study-defined MACE. Secondary objectives were to evaluate all-cause mortality, cardiovascular or cardiac mortality, angiographic no-reflow or slow-flow, and post-contrast renal injury, synthesise discriminatory performance estimates where available, and assess risk of bias and certainty of evidence using prognostic-factor-specific methods.

## Methods

### Protocol and registration

This systematic review and meta-analysis was reported in accordance with the Preferred Reporting Items for Systematic Reviews and Meta-Analyses (PRISMA) 2020 statement. The review protocol was prospectively registered with the International Prospective Register of Systematic Reviews (PROSPERO) on 25 April 2026 under registration number CRD420261378751. No major deviations from the registered protocol occurred; analyses that were not feasible because of sparse data are reported transparently.

### Eligibility criteria

Eligibility criteria were specified *a priori* in the registered protocol and structured according to the PECOS framework, addressing population, exposure or prognostic factor, comparator, outcomes, and study design.

#### Population

We included studies enrolling adult patients (aged ≥18 years) with a confirmed diagnosis of acute coronary syndrome (ACS), encompassing ST-elevation myocardial infarction (STEMI), non-ST-elevation myocardial infarction (NSTEMI), and unstable angina, in whom percutaneous coronary intervention (PCI) was performed during the index ACS episode or index hospitalisation. ACS diagnosis was required to be based on contemporary clinical guideline criteria as reported by the original studies. Studies enrolling mixed populations were eligible only if outcome data for the ACS-with-PCI subgroup could be extracted separately from the original publication or **Supplementary Materials**, or obtained by contacting the study authors. Studies were excluded if they enrolled patients with stable coronary artery disease, conservatively managed ACS without revascularisation, isolated diagnostic coronary angiography without intervention, coronary artery bypass grafting as the index revascularisation strategy, peripheral vascular interventions, or paediatric populations. When a study included both PCI and CABG arms, only the PCI arm was considered.

#### Exposure/prognostic factor

The prognostic factor of interest was the pan-immune-inflammation value (PIV), a composite peripheral blood-derived biomarker integrating neutrophil, platelet, monocyte, and lymphocyte counts (11). PIV was calculated as PIV = (neutrophil count × platelet count × monocyte count)/lymphocyte count, with cell counts expressed as 10^9^/L or equivalent units. Eligible studies were required to report PIV measured at hospital admission, before PCI, or during the early index hospitalisation period before outcome occurrence, as specified by the original study. When both pre-PCI and post-PCI PIV values were reported, admission or pre-PCI values were preferentially extracted. Studies measuring PIV exclusively at later follow-up time points, such as post-discharge visits, were excluded from quantitative synthesis and summarised narratively if relevant. Both categorical and continuous formulations of PIV were eligible, including receiver operating characteristic-derived cut-offs, median splits, tertile or quartile groupings, and continuous modelling as per-unit, per-standard deviation, or per-log increase. The exact cut-off value, derivation method, timing of measurement, and unit of reporting were extracted for each study and considered in subgroup or sensitivity analyses when sufficient data were available.

#### Comparator

For categorical analyses, the comparator was the lowest or reference PIV category as defined in each study. For continuous analyses, effect estimates per-unit, per-standard deviation, or per-log increase were extracted as reported.

#### Outcomes

The primary outcome was study-defined composite major adverse cardiovascular events (MACE) during follow-up. MACE was extracted according to the definition used in each original study, and its components were recorded in detail. Commonly reported components included all-cause death, cardiac death, recurrent myocardial infarction, target vessel or target lesion revascularisation, stroke, heart failure readmission, or other cardiovascular adverse events. Secondary outcomes included all-cause mortality, cardiovascular or cardiac mortality, angiographic no-reflow or slow-flow, post-contrast renal injury, heart failure readmission, recurrent myocardial infarction, target vessel or target lesion revascularisation, and stroke.

#### Study design

Eligible study designs included prospective, retrospective, or registry-based cohort studies reporting associations between PIV and clinical outcomes. Reviews, editorials, case reports, case series without prognostic effect estimates, animal studies, conference abstracts without sufficient data, and duplicate reports from overlapping populations were excluded.

### Information sources and search strategy

A comprehensive literature search was performed in six electronic databases: PubMed/MEDLINE, Embase, Web of Science Core Collection, the Cochrane Library/CENTRAL, Scopus, and CNKI, from database inception to 25 April 2026. To identify ongoing, unpublished, or difficult-to-locate records, we additionally screened ClinicalTrials.gov, the World Health Organization International Clinical Trials Registry Platform (WHO ICTRP), and Google Scholar, with screening limited to the first 200 records sorted by relevance (19). The reference lists of all included studies and relevant reviews were hand-searched. No restrictions were applied regarding language or publication status. The search strategy was developed by the review team and adapted for each database, combining controlled vocabulary terms (MeSH and Emtree) with free-text keywords covering three concept blocks: pan-immune-inflammation value, acute coronary syndrome, and percutaneous coronary intervention. Full search strategies for each database are provided in **Supplementary Table 1**.

### Study selection

All retrieved records were imported into EndNote 21 for deduplication, and the deduplicated dataset was then transferred to Rayyan for screening (20). Two reviewers independently screened titles and abstracts against the predefined eligibility criteria, followed by independent full-text assessment of all potentially eligible records. Disagreements at either stage were resolved through discussion and consensus. Reasons for exclusion at full-text assessment and details of reports not retrieved are presented in **Supplementary Tables 2A, B**, respectively. The study selection process was documented using a PRISMA 2020 flow diagram. Inter-rater agreement during title and abstract screening was assessed using Cohen’s kappa coefficient (21).

### Data extraction

A standardised data extraction form was developed in Microsoft Excel and pilot-tested on three eligible studies before full data extraction. Two reviewers independently extracted data from each included study, and discrepancies were resolved through discussion and consensus. The extracted information covered five domains. (i) Study characteristics: first author, publication year, country, study design, single- versus multicentre setting, recruitment period, sample size, and follow-up duration. (ii) Population characteristics: age, sex, ACS subtype distribution, major cardiovascular risk factors and comorbidities, left ventricular ejection fraction, renal function, Killip class, and key procedural or pharmacological variables. (iii) PIV-related data: calculation formula, units of cell counts, timing of blood sampling, cut-off derivation method, cut-off value, categorisation scheme, and, for continuous analyses, the unit of measurement and any transformation applied. (iv) Outcome data: type of outcome, study-specific MACE definition and components, number of events, follow-up duration, and method of outcome ascertainment. Detailed MACE components and study-specific labels for post-contrast renal injury (CA-AKI, CI-AKI, CIN, and PCAKI) are presented in **Supplementary Table 3**. (v) Effect estimates: adjusted hazard ratios (HRs) with 95% confidence intervals, unadjusted estimates when available, the list of adjustment variables, and the corresponding analytic model. When multiple adjusted models were reported, the most fully adjusted model was preferentially extracted. Adjusted HRs were prioritised over unadjusted estimates. HRs, odds ratios, and risk ratios were extracted separately and were not combined within the same meta-analysis. When essential data were missing or ambiguous, corresponding authors were contacted by email; if no response was received within four weeks, available data were used as reported.

### Risk of bias assessment

Risk of bias in included studies was independently assessed by two reviewers using the Quality in Prognosis Studies (QUIPS) tool, which is designed for prognostic factor research (22). Each study was evaluated across six domains: study participation, study attrition, prognostic factor measurement, outcome measurement, study confounding, and statistical analysis and reporting. Each domain was rated as low, moderate, or high risk of bias based on the signalling questions and guidance provided in the QUIPS framework. An overall judgement of risk of bias was assigned with particular emphasis on the domains of prognostic factor measurement, outcome measurement, study confounding, and statistical analysis and reporting, which are most directly relevant to the validity of associations between PIV and adverse cardiovascular outcomes. Disagreements were resolved through discussion and consensus. Domain-level judgements and supporting rationales for each included study are presented in **Supplementary Table 4**.

### Certainty of evidence assessment

The certainty of evidence for the primary and key secondary outcomes was independently rated by two reviewers using the Grading of Recommendations Assessment, Development and Evaluation (GRADE) approach, adapted for prognostic factor systematic reviews (23, 24). Certainty was evaluated for the primary outcome (study-defined MACE) and key secondary outcomes, including all-cause mortality, cardiovascular or cardiac mortality, no-reflow or slow-flow, and post-contrast renal injury. The assessment considered five domains for downgrading — risk of bias, inconsistency, indirectness, imprecision, and publication bias — and three domains for upgrading — magnitude of association, dose-response gradient, and plausible residual confounding that would reduce the observed association. Each outcome was assigned an overall certainty rating of high, moderate, low, or very low. Disagreements were resolved through discussion and consensus. The complete GRADE evidence profile, including outcome-level certainty ratings and domain-level judgements, is presented in **Supplementary Table 5**.

### Data synthesis and statistical analysis

Quantitative synthesis was performed when at least two studies reported the same outcome with a comparable effect measure; otherwise, findings were summarised narratively. The primary effect measure was the multivariable-adjusted hazard ratio (HR), pooled on the natural logarithmic scale using the standard error derived from the reported 95% confidence interval. When standard errors were not directly reported, they were calculated from confidence intervals or P values using standard formulas (25). When studies reported only Kaplan–Meier curves without HRs, corresponding authors were contacted to obtain numerical estimates; in the absence of usable HR data, such studies were summarised narratively. Hazard ratios reported on different scales, including per-unit, per-standard deviation, and per-log increase, were not converted across scales unless the underlying units and transformation methods were sufficiently consistent for valid standardisation.

The primary synthesis focused on multivariable-adjusted HRs for the association between higher PIV and study-defined MACE. Categorical estimates comparing the highest versus lowest or reference PIV category were pooled as the main categorical analysis, whereas continuous PIV effect estimates were synthesised separately. Odds ratios and risk ratios were pooled separately from HRs and were not combined within the same meta-analysis. Random-effects models were fitted using restricted maximum likelihood (REML), with Hartung–Knapp adjustment for confidence intervals where appropriate (26, 27).

Between-study heterogeneity was quantified using the I² statistic, Cochran’s Q test, and the between-study variance τ² (28). I² values were interpreted descriptively rather than mechanically, as random-effects models were prespecified. Formal subgroup analyses and meta-regression were not performed because the number of studies available for each outcome was limited; clinical and methodological diversity across the prespecified factors (ACS subtype, geographical region, follow-up duration, PIV cut-off derivation method, and MACE definition) was therefore described narratively. Sensitivity analyses included leave-one-out analysis, exclusion of studies at high overall risk of bias, exclusion of single-centre studies, exclusion of studies contributing only unadjusted estimates, exclusion of studies with overlapping populations, re-analysis using a fixed-effect model, and exploratory comparisons by study design or single- versus multicentre status.

Publication bias and small-study effects were assessed visually using funnel plots and statistically using Egger’s regression test when at least 10 studies were available for a given outcome (29, 30). Trim-and-fill analysis was considered as an exploratory assessment when appropriate. All analyses were conducted in R version 4.6.0 using the meta and metafor packages, with two-sided P values and 95% confidence intervals reported throughout.

## Results

### Study selection

**Figure 1:**
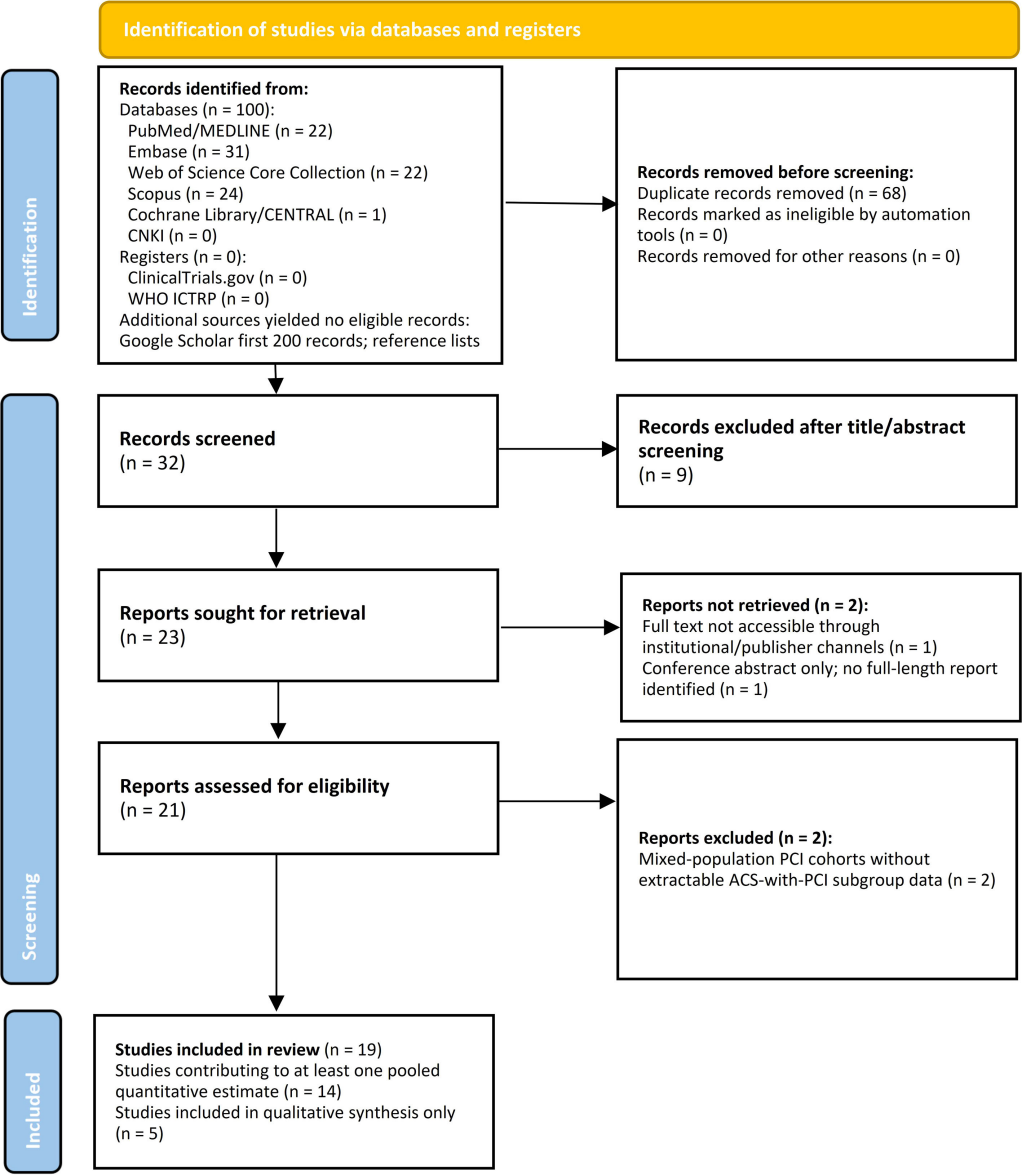
PRISMA 2020 flow diagram of study identification, screening, and inclusion. Database searches identified 100 records from PubMed/MEDLINE, Embase, Web of Science Core Collection, Scopus, the Cochrane Library/CENTRAL, and CNKI; trial registry searches identified no additional records. After removal of 68 duplicates, 32 records were screened, nine were excluded, and 23 reports were sought for full-text retrieval. Two reports could not be retrieved, and 21 full-text reports were assessed for eligibility, of which two were excluded with reasons. Overall, 19 studies were included in the systematic review; 14 contributed to at least one pooled quantitative estimate and five were included in the qualitative synthesis only. Screening of Google Scholar and hand-searching of reference lists yielded no additional eligible studies. ACS, acute coronary syndrome; CNKI, China National Knowledge Infrastructure; ICTRP, International Clinical Trials Registry Platform; PCI, percutaneous coronary intervention; PIV, pan-immune-inflammation value; PRISMA, Preferred Reporting Items for Systematic Reviews and Meta-Analyses; WHO, World Health Organization.

The electronic database searches identified 100 records, including 22 from PubMed, 31 from Embase, 22 from Web of Science Core Collection, 24 from Scopus, one from the Cochrane Library/CENTRAL, and none from CNKI. Searches of trial registries (ClinicalTrials.gov and the WHO International Clinical Trials Registry Platform), screening of the first 200 Google Scholar results sorted by relevance, and hand-searching of reference lists from included studies and relevant reviews yielded no additional eligible studies. After removal of 68 duplicate records, 32 unique records underwent title and abstract screening. Nine records were excluded at this stage, and 23 reports were sought for full-text retrieval. Of these, two reports could not be retrieved despite attempts through institutional library access, journal websites, and e-mail contact with the corresponding authors, including one conference abstract for which no full-length report was identified. Details and retrieval attempts for these reports are provided in **Supplementary Table 2B**. Twenty-one full-text reports were assessed for eligibility, of which two were excluded; reasons for exclusion are listed in **Supplementary Table 2A**. Overall, 19 studies were included in the systematic review, of which 14 contributed to at least one pooled quantitative estimate and five were included in the qualitative synthesis only because their outcomes or effect measures were not compatible with any pooled analysis, or because extractable PIV-specific adjusted effect estimates or PIV-only discrimination metrics were unavailable. Inter-rater agreement during title and abstract screening was almost perfect, with a Cohen’s kappa coefficient of 0.86. The study selection process is summarised in **Figure 1**.

### Study characteristics

A total of 19 studies, including 18,715 patients with acute coronary syndrome (ACS) undergoing percutaneous coronary intervention (PCI) in the analytic PIV cohorts, were included in this systematic review (**Table 1**). Studies were conducted in Türkiye (n = 10), mainland China (n = 7), Taiwan (n = 1), and South Korea (n = 1). The majority were retrospective cohort studies (n = 16), of which 15 were single-centre and one explicitly reported a multicentre design (42); the remaining three were prospective single-centre cohort studies (12, 34, 37). Among studies reporting recruitment periods, recruitment spanned from January 2013 to March 2025, and individual study sample sizes ranged from 143 to 2,576 participants, the latter representing the combined derivation and validation cohorts of Genc et al., 2025 (39).

**Table 1:** Characteristics of included studies.

#	Study	Country	Design	Recruitment period	N	Age, years	Male, %	ACS subtype	PIV timing	PIV cut-off (derivation)	Primary outcome	Follow-up
1	Çiçek et al., 2026 (31)	Türkiye	Retrospective, single-center	NR	2,325	54.87 ± 12.04 / 58.03 ± 14.51 by CA-AKI status	84.9 / 80.1 by CA-AKI status	STEMI 100%	Admission/pre-PCI	>320 (ROC-derived)	CA-AKI	48–72 h post-PCI
2	Keskin et al., 2026 (32)	Türkiye	Retrospective, single-center	01/2023–07/2023	329	62.57 ± 12.12	79.9	STEMI 100%	Admission	NR (ML feature)	Long-term all-cause mortality	24 months
3	Qu et al., 2026 (33)	China	Retrospective, single-center	01/2022–12/2023	200	61.62 ± 2.39 / 59.46 ± 2.60 by MACE status	52.0	STEMI 100%	Admission	223.04 (ROC-derived)	MACE	12 months
4	Ma et al., 2025 (34)	China	Prospective, single-center	11/2023–12/2024	772 (799 enrolled)	68 ± 6	73	STEMI 100% (age ≥60 years)	First 12 h after admission	693 (ROC-derived)	In-hospital heart failure	In-hospital
5	Chen et al., 2025 (35)	China	Retrospective, single-center	11/2023–03/2025	143 (MI subgroup; 263 UAP cohort)	63.29 ± 7.48 / 62.83 ± 7.99 by MACE status	74.8	MI subgroup post-PCI	Baseline	389.37 (ROC-derived)	MACE	Median 12 months
6	Konte et al., 2025 (36)	Türkiye	Retrospective, single-center	05/2022–07/2024	317	60.09 ± 12.74	78.23	STEMI/NSTEMI: 66.25%/33.75%	Baseline/pre-intervention	>621.7 × 10^6 (ROC-derived)	CI-AKI	48–72 h post-contrast
7	Xu et al., 2025 (37)	China	Prospective, single-center	12/2023–12/2024	184	59.16 ± 12.81 / 65.26 ± 14.54 by MACE status	81.0	STEMI 100%	Within 24 h of symptom onset	LnPIV 24 h (continuous/ROC)	MACE (CEC-adjudicated)	6 months
8	Ting et al., 2025 (14)	Taiwan	Retrospective, single-center	01/2013–12/2022	689	61.2 ± 12.4 / 72.8 ± 14.7 by 1-year mortality status	83.2	STEMI 100%	Initial CBC	>251.769 (ROC-derived)	1-year all-cause mortality	1 year (primary); median 4.7 years (total)
9	Liu et al., 2025 (12)	China	Prospective, single-center	01/2016–12/2018	1,360	NR (age ≥65 years reported)	74.8	AMI/UA: 61.3%/38.8%	Admission	≥355.79 (ROC-derived)	MACEs	Median 1,006 days (~33 months)
10	Byoun et al., 2025 (15)	South Korea	Retrospective, single-center	2014–2020	1,651 (1,606 with 1-year outcomes)	65.9 ± 10.3 / 66.9 ± 11.5 by PIV group	67.4	UA/NSTEMI: 80.9%/19.1%	Baseline	>256.3 (ROC-derived)	MACEs	1 year; median 400 days
11	Ömür et al., 2024 (38)	Türkiye	Retrospective	08/2017–08/2023	1,693	70.2 ± 9.85 / 70.84 ± 9.85 by CIN status	51.5	UA/NSTEMI/STEMI mixed	Admission/pre-procedural	532.27 (ROC-derived)	CIN	48 h post-contrast
12	Genc et al., 2025 (39)	Türkiye	Retrospective, single-center	01/2022–11/2023	1,546 (derivation) + 1,030 (validation)	59.6 ± 12.5 / 59.3 ± 13.0 by cohort	79.0 / 78.4 by cohort	STEMI 100%	Admission	NR (discrimination model)	In-hospital all-cause mortality	In-hospital
13	Yang et al., 2024 (40)	China	Retrospective	2016–2023	542	64.13 ± 14.42 / 59.73 ± 13.29 by MACE status	80.3	STEMI 100%	Admission	793.755 (ROC-derived); LnPIV in models	In-hospital MACE	In-hospital with 1-week follow-up
14	Kurtul and Gok, 2024 (18)	Türkiye	Retrospective, single-center	06/2019–12/2022	839	60.7 ± 12.9	68.9	ACS-PCI with DES	Preinterventional	>576 (ROC-derived)	PCAKI	Within 72 h post-PCI
15	Şen et al., 2024 (17)	Türkiye	Retrospective, single-center	2019–2023	687	61 ± 12	77.6	STEMI 100%	Baseline/pre-pPCI	≥804 (ROC-derived)	No-reflow / impaired coronary flow	Immediate post-pPCI
16	Liu et al., 2023 (13)	China	Retrospective, single-center	01/2016–01/2020	216	median 63 (IQR 51–69)	83.8	STEMI-PCI	Admission (per protocol hierarchy)	306.25 (admission ROC-derived)	MACEs	Within 1 year after discharge
17	Bayramoğlu and Hidayet, 2023 (16)	Türkiye	Retrospective, single-center	07/2020–07/2022	1,212	57.5 ± 12.1 / 61.8 ± 11.7 by no-reflow status	82.5 / 87.0 by no-reflow status	STEMI 100%	Baseline before intervention	≥889 (ROC-derived)	No-reflow (TIMI ≤2)	Immediate post-PCI
18	Murat et al., 2023 (42)	Türkiye	Retrospective, multi-center	2018–2022	658	58.7 ± 17.1	76.9	STEMI 100%	Admission before PCI	906.14 (ROC-derived)	All-cause mortality	Mean 18.86 ± 8.67 months
19	Zorlu et al., 2025 (41)	Türkiye	Retrospective, single-center	01/2018–01/2024	2,367	66.27 ± 1.12 / 67.23 ± 3.02 by CIN status	51.0	STEMI 100%	Pre-procedural	548 (ROC-derived)	CIN	48–72 h post-PCI

For each study, the table presents the first author and year of publication, country, study design, recruitment period, analytic sample size, age, sex distribution, acute coronary syndrome (ACS) subtype composition, timing of pan-immune-inflammation value (PIV) measurement, PIV cut-off and method of derivation, the primary outcome assessed in the original study, and follow-up duration. Continuous variables are presented as mean ± standard deviation or median with interquartile range or range, as reported in the original publication. When studies reported baseline characteristics stratified by outcome status (e.g., MACE versus no MACE; CA-AKI versus no CA-AKI) or by PIV group, both values are shown separated by a slash, with the stratification variable indicated. Sample sizes correspond to the cohort with PIV measurement and outcome data available; where the analytic cohort was reduced from the originally enrolled population through subsequent eligibility filtering, the analytic sample size is presented first and the originally enrolled or parent cohort is shown in parentheses. For Genc et al. (39), the derivation and validation cohorts are reported separately. PIV cut-offs derived using receiver operating characteristic (ROC) curve analysis are presented with the specific cut-off value and the term "ROC-derived"; one study (37) modelled PIV as a continuous variable on the natural logarithmic scale (LnPIV), and two studies (32, 39) did not report a discrete PIV cut-off because PIV was used as a candidate feature in a machine-learning model or as a component of a multivariable discrimination score, respectively. When more than one time point of PIV measurement was reported, the admission or pre-procedural value was preferentially extracted in accordance with the prespecified measurement hierarchy specified in the registered review protocol (PROSPERO CRD420261378751). Detailed study-specific definitions and components of the composite cardiovascular outcomes are presented in **Supplementary Table S3**, and the corresponding risk-of-bias judgments using the Quality in Prognosis Studies (QUIPS) tool are presented in **Supplementary Table S4**.

ACS, acute coronary syndrome; AMI, acute myocardial infarction; CA-AKI, contrast-associated acute kidney injury; CBC, complete blood count; CEC, Clinical Events Committee; CI-AKI, contrast-induced acute kidney injury; CIN, contrast-induced nephropathy; DES, drug-eluting stent; HF, heart failure; IQR, interquartile range; LnPIV, natural logarithm of pan-immune-inflammation value; MACE, major adverse cardiovascular events; MI, myocardial infarction; ML, machine learning; NR, not reported; NSTEMI, non-ST-elevation myocardial infarction; PCAKI, post-contrast acute kidney injury; PCI, percutaneous coronary intervention; PIV, pan-immune-inflammation value; pPCI, primary percutaneous coronary intervention; ROC, receiver operating characteristic; SD, standard deviation; STEMI, ST-elevation myocardial infarction; UA, unstable angina; UAP, unstable angina pectoris.

The mean age of participants ranged from approximately 57 to 70 years across studies, with three studies enrolling specifically older populations (34: ≥60 years; 12 : ≥65 years; 38: mean 70.2 years). Most cohorts were predominantly male, with the proportion of male participants ranging from approximately 51% to 87% and clustering between 70% and 85% in the majority of studies. With respect to ACS subtype, 13 studies enrolled patients with ST-elevation myocardial infarction (STEMI) exclusively, whereas the remaining six studies enrolled mixed ACS populations comprising STEMI, non-ST-elevation myocardial infarction (NSTEMI), and/or unstable angina, with subtype proportions varying considerably across studies.

In most studies, PIV was measured at hospital admission or before PCI. Two studies specified distinct early measurement windows: within the first 12 hours of admission in Ma et al. (34) and within 24 hours of symptom onset in Xu et al. (37). All studies measured PIV before outcome occurrence. Sixteen studies derived a discrete PIV cut-off using receiver operating characteristic (ROC) curve analysis, with numeric cut-off values ranging from 223.04 to 906.14, representing more than fourfold variation. This range likely reflected differences in study populations, outcome definitions, and cut-off derivation methods, and the reported thresholds should be interpreted descriptively rather than as directly interchangeable clinical thresholds. One study (37) modelled PIV primarily as a continuous variable on the natural logarithmic scale, whereas two studies did not report a discrete cut-off because PIV was used as a candidate feature in a machine-learning model (32) or as a component of a multivariable discrimination score (39).

The principal extractable outcomes varied across studies. Seven studies reported a study-defined composite major adverse cardiovascular event (MACE) endpoint, three reported long-term all-cause mortality, one reported in-hospital all-cause mortality, one reported in-hospital heart failure, five reported post-contrast renal injury outcomes variably labelled as CA-AKI, CI-AKI, CIN, or PCAKI, and two reported angiographic no-reflow or slow-flow. Follow-up duration ranged from immediate post-procedural assessment and 48–72-hour creatinine windows for renal and angiographic outcomes to a median of 1,006 days, approximately 33 months, for long-term cardiovascular outcomes (12); the longest reported median follow-up was 4.7 years (14). Detailed study-specific definitions and components of the composite cardiovascular outcomes are presented in **Supplementary Table 3**.

### Risk of bias within studies

**Figure 2:**
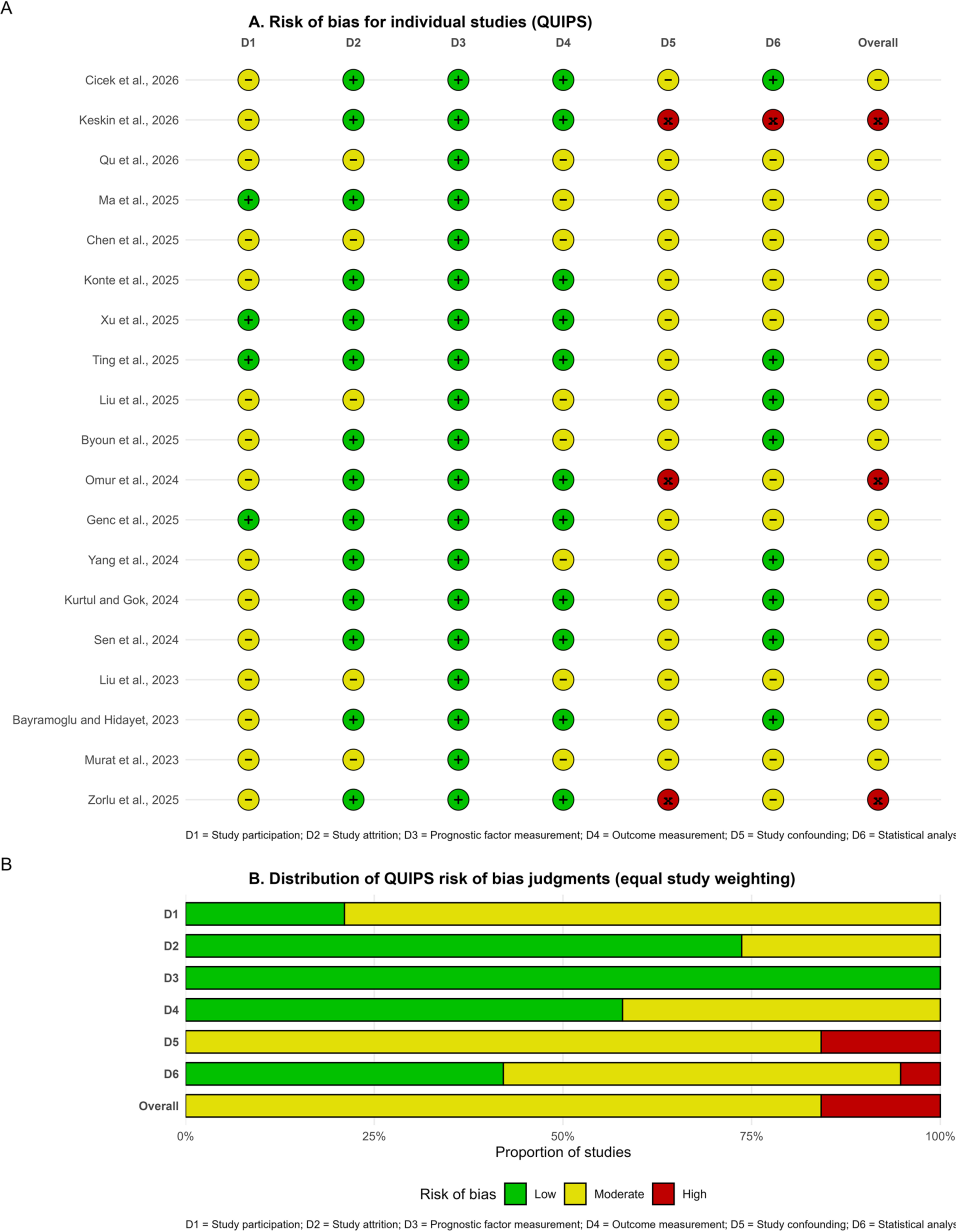
Risk of bias of the included studies assessed using the quality in prognosis studies (QUIPS) tool. **(A)** Domain-level and overall risk-of-bias judgements for each of the 19 included studies. **(B)** Distribution of risk-of-bias judgements across studies for each domain; each study contributed equally to the summary. Risk of bias was independently assessed by two reviewers, with disagreements resolved through discussion and consensus. Domains: D1 = study participation; D2 = study attrition; D3 = prognostic factor measurement; D4 = outcome measurement; D5 = study confounding; D6 = statistical analysis and reporting. Symbols: + = low risk; − = moderate risk; × = high risk. Detailed domain-level judgements and supporting rationales for each study are provided in **Supplementary Table 4.3.4** Primary outcome: study-defined major adverse cardiovascular events.

Risk of bias of the 19 included studies, assessed independently by two reviewers using the QUIPS tool with disagreements resolved through discussion and consensus, is summarised in **Figure 2**; domain-level judgements and supporting rationales are presented in **Supplementary Table 4**. Overall, 16 studies were rated as having moderate risk of bias and three as having high risk of bias; none was rated as low risk.

Prognostic factor measurement was generally judged to be at low risk of bias, because PIV was derived from routine complete blood count parameters using the standard PIV formula and was measured at admission or in the early pre-procedural window in most studies. Outcome measurement was also predominantly rated at low or moderate risk of bias, with more rigorous ascertainment in studies linking mortality to national death registry records (14) or employing blinded clinical event committee adjudication (37). Moderate judgements were most often related to incomplete reporting of follow-up completeness, residual confounding, and variable adjustment for procedural or pharmacological factors.

Three studies were rated at high overall risk of bias (32, 38, 41). These ratings reflected the absence of a PIV-specific multivariable adjusted prognostic model, insufficient adjustment for confounding, or concerns regarding statistical analysis and reporting. Keskin et al. (32) used PIV as a candidate feature within a machine learning framework rather than as a covariate in a standalone adjusted prognostic model, whereas Ömür et al. (38) and Zorlu et al. (41) focused on receiver operating characteristic discrimination and biomarker combinations without fitting a PIV-specific multivariable model. These findings indicate that interpretation of the pooled estimates should account for residual confounding and limitations in statistical reporting.

### Primary outcome: study-defined major adverse cardiovascular events

Two evidence streams informed the association between PIV and study-defined MACE: multivariable-adjusted hazard ratios (HRs) comparing higher versus lower PIV categories, and discriminatory performance estimates expressed as receiver operating characteristic curve areas under the curve (AUCs). Hartung–Knapp adjustment was not applied to the adjusted HR analysis because only two studies contributed to the pooled estimate; for the AUC analysis, random-effects pooling was performed on the logit-transformed AUC scale with Hartung–Knapp adjustment according to the prespecified analysis plan.

**Figure 3:**
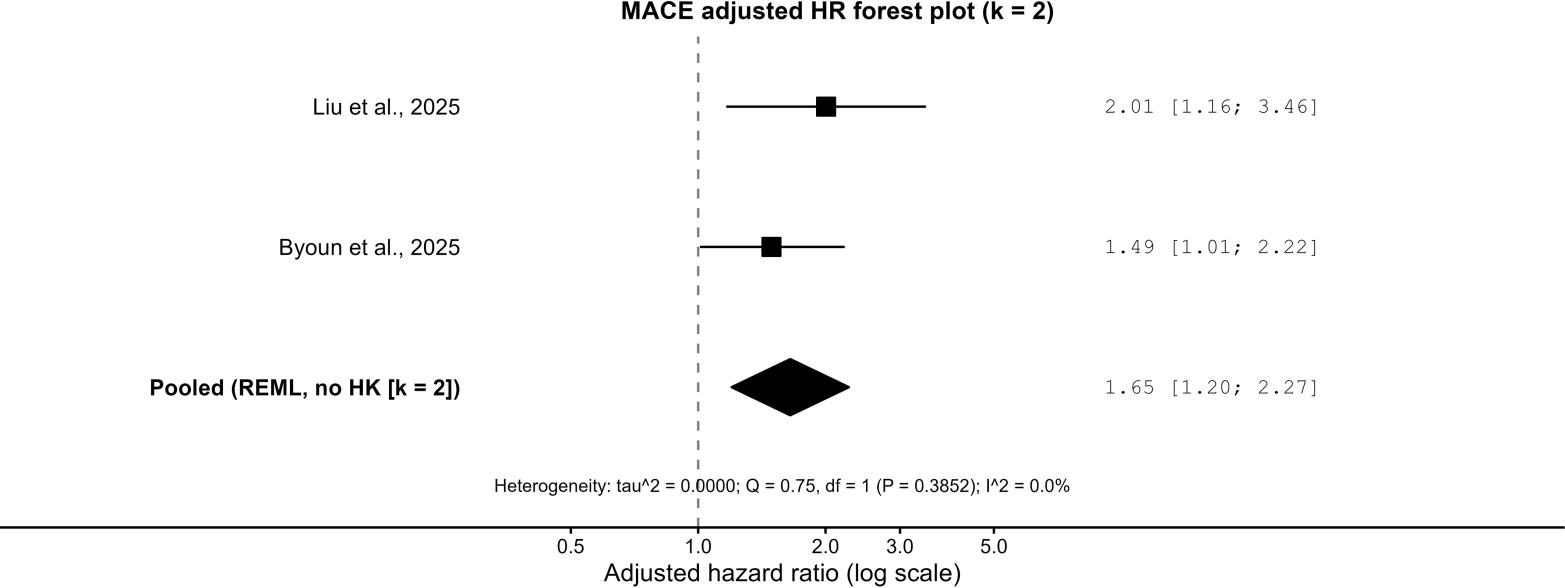
Forest plot of multivariable-adjusted hazard ratios comparing higher versus lower pan-immune-inflammation value categories for study-defined major adverse cardiovascular events in patients with acute coronary syndrome undergoing percutaneous coronary intervention. Estimates were pooled using random-effects meta-analysis with restricted maximum likelihood estimation of the between-study variance on the natural logarithmic scale. Hartung–Knapp adjustment was not applied owing to the small number of contributing studies (k = 2). Squares represent study-level adjusted hazard ratios, with horizontal lines indicating 95% confidence intervals; the diamond represents the pooled estimate. CI, confidence interval; HR, hazard ratio; MACE, major adverse cardiovascular events; PIV, pan-immune-inflammation value.

Two studies (12, 15; combined n = 2,966; events = 169) provided multivariable-adjusted HRs comparing higher versus lower PIV categories. Pooling these estimates yielded a multivariable-adjusted HR of 1.65 (95% CI 1.20 to 2.27) for the association between higher PIV and study-defined MACE, with no observed statistical heterogeneity (I² = 0.0%). The two contributing estimates were directionally concordant: Liu et al. (12) reported an adjusted HR of 2.01 (95% CI 1.16 to 3.46), and Byoun et al. (15) reported an adjusted HR of 1.49 (95% CI 1.01 to 2.22). Because this estimate was based on only two studies, its generalisability remains limited. Forest plot details are presented in **Figure 3**.

Five studies ((12, 13, 33, 35, 37 combined n = 2,103; events = 389) reported AUCs for the discrimination of study-defined MACE by PIV. For Liu et al., 2023 (13), admission PIV was extracted according to the prespecified measurement hierarchy. Pooling on the logit-transformed AUC scale yielded a pooled AUC of 0.729 (95% CI 0.578 to 0.841), reflecting moderate overall discrimination; however, statistical heterogeneity was substantial (I² = 83.0%), and the pooled AUC should therefore be interpreted cautiously as an exploratory summary. This heterogeneity likely reflects clinical and methodological diversity across studies, including differences in follow-up duration, the components of the study-defined MACE composite, and the timing of PIV measurement. Forest plot details for the AUC pooling are presented in **Supplementary Figure 6**. The certainty of evidence for the primary MACE outcome was rated as low overall, downgraded for risk of bias and inconsistency in the discrimination evidence (**Table 2**, **Supplementary Table 5**).

**Table 2:** GRADE summary of findings for PIV and adverse cardiovascular outcomes after ACS treated with PCI.

Outcome	No. of studies (participants; events)	Pooled effect estimate (95% CI)	Certainty of evidence (GRADE)	Plain-language interpretation
Study-defined MACE after ACS treated with PCI	Adjusted association: k = 2 (n = 2,966; events = 169). Predictive performance: k = 5 (n = 2,103; events = 389).	Adjusted HR 1.65 (95% CI 1.20-2.27; I² = 0%). AUC 0.729 (95% CI 0.578 to 0.841; I² = 83.0%).	Low. Downgraded for risk of bias and inconsistency, mainly due to moderate QUIPS concerns and substantial heterogeneity in AUC estimates.	Higher PIV was associated with increased risk of study-defined MACE. PIV showed moderate discriminatory performance, but heterogeneity limits confidence in its standalone clinical use.
All-cause mortality after ACS treated with PCI	Adjusted association: k = 2 (n = 2,295; events = 97). Predictive performance: single AUC study with CI (n = 689; events = 61).	Adjusted HR 3.51 (95% CI 2.15-5.74; I² = 0%). k=2 n=2,295 events=97; one additional study reported PIV-specific AUCs without 95% CIs and was excluded from AUC pooling.	Moderate. Downgraded mainly for imprecision and limited evidence base; no high-risk QUIPS studies contributed to the compatible HR subset.	Higher PIV was consistently associated with higher all-cause mortality in the compatible adjusted analyses. The association appears clinically important, but the number of studies remains small.
Cardiovascular or cardiac mortality after ACS treated with PCI	Single component estimate from Byoun et al. (15): k = 1 (n = 1,606; events = 24).	Adjusted HR 3.24 (95% CI 1.34-7.81).	Low. Downgraded for very serious imprecision because evidence came from one Byoun 2025 component-level estimate with few cardiac-death events and a wide CI.	Higher PIV may be associated with increased cardiac mortality, but this finding is based on one component-level estimate from Byoun et al. (15). Liu et al. (12) reported cardiogenic mortality only within composite MACE and did not provide a separately extractable PIV-specific component effect.
No-reflow or slow-flow after primary PCI	Predictive performance: k = 2 (n = 1,899; events = 262). Association estimates were not pooled because exposure scaling differed.	AUC 0.828 (95% CI 0.808-0.846; I^2^ = 0.0%).	Moderate. Downgraded for imprecision because the evidence base was limited to two retrospective STEMI pPCI studies.	PIV showed good and consistent discrimination for no-reflow or slow-flow. External validation is still needed before using PIV as a decision tool for angiographic risk.
Contrast-associated AKI / contrast-induced nephropathy after PCI	Predictive performance: k = 5 (n = 7,541; events = 1,996). Exploratory adjusted OR subset: k = 2 (n = 3,164; events = 617).	AUC 0.771 (95% CI 0.617-0.875; I^2^ = 95.1%). Exploratory adjusted OR 4.91 (95% CI 0.87-27.89; I^2^ = 90.3%).	Very low. Downgraded for risk of bias, inconsistency, and imprecision, including two high-risk QUIPS studies and substantial heterogeneity.	PIV may help identify patients at higher risk of contrast-related AKI, but the certainty is very low. The wide OR CI and high heterogeneity mean the magnitude of risk remains uncertain.

*Patient or population:* adults (≥18 years) with acute coronary syndrome (ST-elevation myocardial infarction, non-ST-elevation myocardial infarction, or unstable angina) undergoing percutaneous coronary intervention during the index hospitalization. *Prognostic factor:* pan-immune-inflammation value (PIV) measured at hospital admission, before percutaneous coronary intervention, or during the early index hospitalization period. *Comparator:* lowest or reference PIV category for categorical analyses; per-unit, per-standard deviation, or per-log increase for continuous analyses. *Setting:* hospital-based cohorts (prospective, retrospective, or registry-based) from multiple countries. The certainty of evidence was rated using the Grading of Recommendations Assessment, Development and Evaluation (GRADE) approach adapted for prognostic factor systematic reviews, considering risk of bias, inconsistency, indirectness, imprecision, and publication bias as downgrading domains, and magnitude of association, dose–response gradient, and plausible residual confounding as upgrading domains. The full evidence profile, including domain-level judgments, is presented in **Supplementary Table S5**. Pooled effect estimates are presented separately for adjusted prognostic association (multivariable-adjusted hazard ratios or odds ratios) and discriminatory performance (area under the receiver operating characteristic curve), as these represent distinct evidence streams that were not combined within the same meta-analysis. Publication bias could not be formally assessed for any outcome because fewer than 10 studies contributed to each analysis. The systematic review was prospectively registered with PROSPERO (CRD420261378751).

AUC, area under the receiver operating characteristic curve; CA-AKI, contrast-associated acute kidney injury; CI, confidence interval; GRADE, Grading of Recommendations Assessment, Development and Evaluation; HR, hazard ratio; *I*², I-squared statistic for between-study heterogeneity; *k*, number of studies; MACE, major adverse cardiovascular events; OR, odds ratio; PCI, percutaneous coronary intervention; PIV, pan-immune-inflammation value; pPCI, primary percutaneous coronary intervention; STEMI, ST-elevation myocardial infarction.

### Secondary outcomes

**Figure 4:**
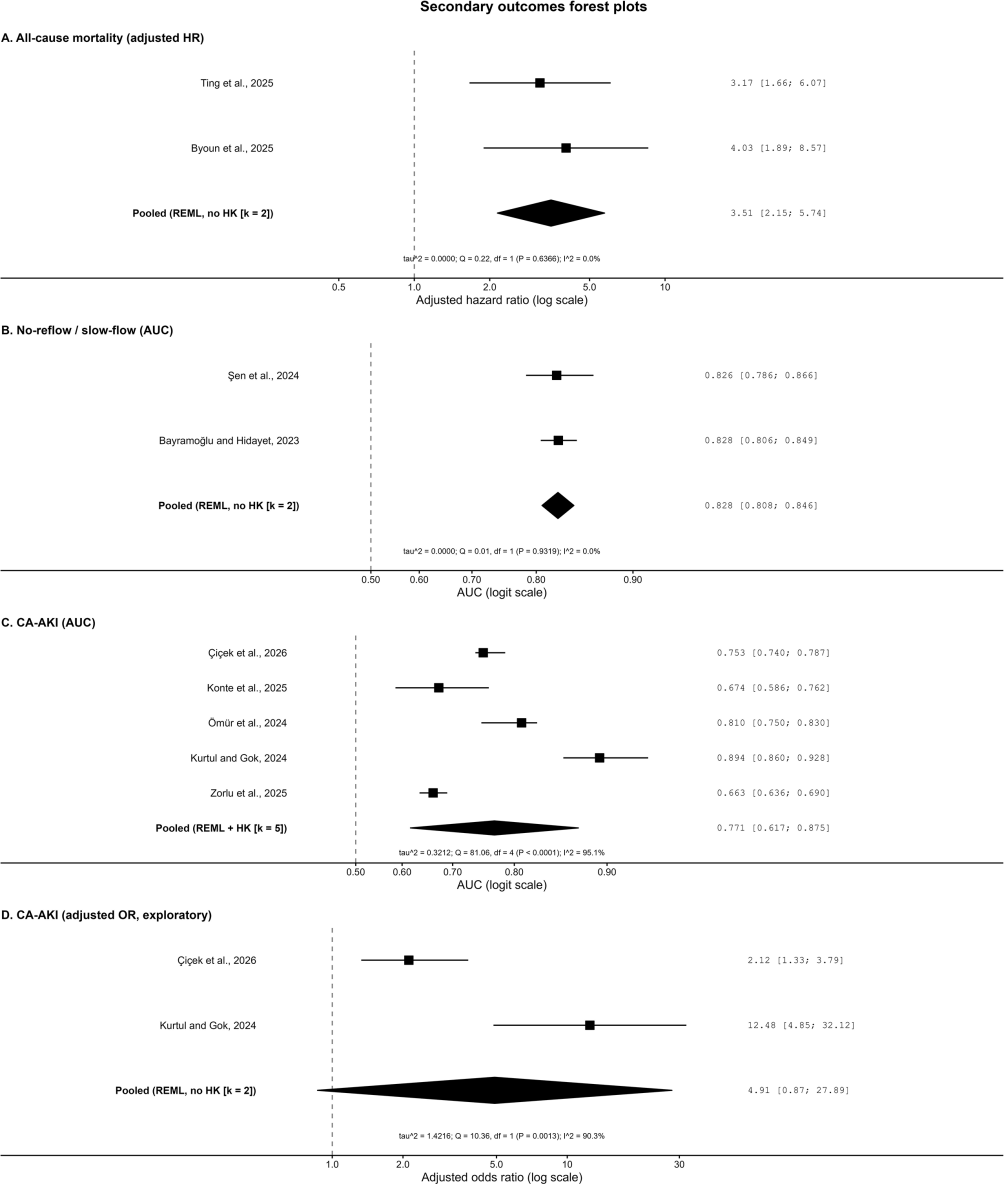
Forest plots of secondary outcomes. **(A)** Multivariable-adjusted hazard ratios for all-cause mortality (k = 2). **(B)** Receiver operating characteristic curve areas under the curve (AUCs) for angiographic no-reflow or slow-flow (k = 2). **(C)** AUCs for post-contrast renal injury (k = 5). **(D)** Multivariable-adjusted odds ratios for post-contrast renal injury (k = 2; exploratory analysis). Hazard and odds ratios were pooled using random-effects meta-analysis with restricted maximum likelihood (REML) estimation on the natural logarithmic scale; AUCs were pooled on the logit-transformed scale. Hartung–Knapp adjustment was applied for the post-contrast renal injury AUC analysis (k = 5) but not for the k = 2 analyses shown in panels **(A, B, D)**. Squares represent study-level estimates with 95% confidence intervals; diamonds represent pooled estimates. The dashed reference lines indicate the null effect for each scale (HR = 1; OR = 1; AUC = 0.50). For panels **(B, C)** the x-axis is displayed on the logit scale with reference labels showing the corresponding AUC values. AUC, area under the receiver operating characteristic curve; CI, confidence interval; HK, Hartung–Knapp; HR, hazard ratio; OR, odds ratio; PIV, pan-immune-inflammation value; REML, restricted maximum likelihood.

For all-cause mortality, two studies (14, 15; combined n = 2,295; events = 97) provided multivariable-adjusted HRs comparing higher versus lower PIV categories. Pooling these estimates yielded an adjusted HR of 3.51 (95% CI 2.15 to 5.74), with no observed statistical heterogeneity (I² = 0.0%); Hartung–Knapp adjustment was not applied owing to the small number of contributing studies (k = 2). The two contributing estimates were directionally concordant: Ting et al., 2025 reported an adjusted HR of 3.17 (95% CI 1.66 to 6.07) for 1-year all-cause mortality after STEMI, and Byoun et al. (15) reported an adjusted HR of 4.03 (95% CI 1.89 to 8.57) for 1-year all-cause death in NSTE-ACS. Genc et al. (39), which was included in the qualitative synthesis only, reported PIV-specific in-hospital all-cause mortality AUCs of 0.586, 0.710, and 0.631 in the derivation, validation, and overall cohorts, respectively, within an mNPS-centred discrimination framework, but did not report 95% confidence intervals or standard errors and did not provide a PIV-specific multivariable adjusted estimate eligible for pooling. Forest plot details for the adjusted HR pooling are presented in **Figure 4A**.

For cardiovascular or cardiac mortality, a single multivariable-adjusted estimate was identified, derived from the component-level analysis of Byoun et al. (15) (n = 1,606; events = 24), reporting an adjusted HR of 3.24 (95% CI 1.34 to 7.81) for 1-year cardiac death. No additional study reported a PIV-specific adjusted estimate for cardiovascular mortality that was extractable separately from a composite endpoint, and quantitative pooling was therefore not performed. The single-study estimate is reported in **Table 2**.

For angiographic no-reflow or slow-flow, two studies (16, 17; combined n = 1,899; events = 262) reported AUCs in retrospective ST-elevation myocardial infarction primary PCI cohorts. Şen et al., 2024 defined the outcome as TIMI 0–2 flow or TIMI 3 flow with myocardial blush <2 (AUC 0.826, 95% CI 0.786 to 0.866), and Bayramoğlu and Hidayet 2023 defined it as final TIMI ≤2 flow assessed by two blinded interventional cardiologists (AUC 0.828, 95% CI 0.806 to 0.849). Pooling on the logit-transformed AUC scale yielded a pooled AUC of 0.828 (95% CI 0.808 to 0.846), with no observed statistical heterogeneity (I² = 0.0%); Hartung–Knapp adjustment was not applied (k = 2). No PIV-specific adjusted association estimates compatible for pooling were reported across these studies. Forest plot details are presented in **Figure 4B**.

For post-contrast renal injury, five studies (18, 31, 36, 38, 41); combined n = 7,541; events = 1,996) reported AUCs. Study-specific labels included CA-AKI, CI-AKI, CIN, and PCAKI, and these outcomes were synthesised under the broader category of post-contrast renal injury, with study-specific definitions retained. Pooling on the logit-transformed AUC scale with Hartung–Knapp adjustment yielded a pooled AUC of 0.771 (95% CI 0.617 to 0.875), with very high statistical heterogeneity (I² = 95.1%). Because of this very high between-study heterogeneity, the pooled AUC should be interpreted with caution and regarded as an exploratory, rather than precise, summary. Individual study AUCs ranged from 0.663 (41) to 0.894 (18), reflecting substantial between-study variability in cut-off derivation, contrast media protocols, and outcome definitions. A prespecified sensitivity analysis excluding the two studies at high overall risk of bias (38, 41) yielded a pooled AUC of 0.790 (95% CI 0.386 to 0.957), with I² = 93.6% across the remaining three studies, indicating persistent heterogeneity and considerable imprecision. Forest plot details for the pooled AUC analysis are presented in **Figure 4C**.

An exploratory pooling of multivariable-adjusted ORs comparing higher versus lower PIV categories for post-contrast renal injury was performed using the two studies that provided compatible categorical adjusted estimates (31: OR 2.12 [95% CI 1.33 to 3.79]; 18: OR 12.48 [95% CI 4.85 to 32.12]; combined n = 3,164; events = 617). The pooled adjusted OR was 4.91 (95% CI 0.87 to 27.89), with very high statistical heterogeneity (I² = 90.3%). This association estimate should be interpreted cautiously because only two studies contributed and between-study heterogeneity was substantial. Forest plot details are presented in **Figure 4D**. The certainty of evidence for the secondary outcomes ranged from very low to moderate, with moderate certainty for all-cause mortality and no-reflow or slow-flow, low certainty for cardiac mortality, and very low certainty for post-contrast renal injury (**Table 2**, **Supplementary Table 5**).

### Subgroup analyses, sensitivity analyses, and assessment of publication bias

Prespecified subgroup analyses according to ACS subtype, geographic region, follow-up duration, PIV cut-off derivation method, and study-defined MACE composition were not performed because the number of studies within each outcome and subgroup stratum was too small to support meaningful analysis. The clinical and methodological diversity underlying these prespecified factors is therefore described narratively in the study characteristics and outcome-specific results, and considered when interpreting the findings.

Sensitivity analyses were performed for the two outcomes with five contributing studies: MACE AUC and post-contrast renal injury AUC. For MACE AUC, leave-one-out re-pooling yielded pooled AUC estimates ranging from 0.677 (95% CI 0.570 to 0.769) to 0.752 (95% CI 0.551 to 0.882). Exclusion of Qu et al. (33) reduced statistical heterogeneity from I² = 83.0% to I² = 49.1%, suggesting that this study was a major contributor to between-study variability. For post-contrast renal injury AUC, leave-one-out re-pooling yielded pooled AUC estimates ranging from 0.731 (95% CI 0.604 to 0.828) to 0.795 (95% CI 0.604 to 0.908). Heterogeneity remained very high across all leave-one-out iterations (I² = 91.4% to 96.2%), indicating that the variability was not driven by any single study. The prespecified sensitivity analysis excluding the two studies at high overall risk of bias for the post-contrast renal injury AUC analysis is reported with the secondary outcome results.

Fixed-effect re-analysis was performed as a methodological sensitivity check. For pooled analyses with no observed heterogeneity, including MACE adjusted HR, all-cause mortality HR, and no-reflow AUC, fixed-effect and random-effects estimates were identical. For MACE AUC and post-contrast renal injury AUC, fixed-effect models produced slightly lower point estimates and substantially narrower confidence intervals than random-effects models. For the exploratory post-contrast renal injury adjusted OR analysis, the fixed-effect estimate was 3.21 (95% CI 2.03 to 5.08), whereas the random-effects estimate was 4.91 (95% CI 0.87 to 27.89), indicating that the fixed-effect model did not adequately reflect uncertainty in the presence of very high heterogeneity (I² = 90.3%). Random-effects estimates were therefore retained as the primary results across analyses.

Formal assessment of publication bias using funnel plot inspection or Egger’s regression test was not performed for any outcome because the prespecified threshold of at least ten contributing studies per outcome was not met. The potential influence of publication bias and small-study effects therefore cannot be excluded and represents an acknowledged limitation of the present synthesis. Detailed leave-one-out and fixed-effect versus random-effects comparison results are presented in **Supplementary Table 7**.

## Discussion

This systematic review and meta-analysis synthesised evidence from 19 cohort studies comprising 18,715 patients with acute coronary syndrome (ACS) undergoing percutaneous coronary intervention (PCI) to evaluate the prognostic and discriminatory value of the pan-immune-inflammation value (PIV) for adverse cardiovascular outcomes. Overall, the evidence suggested an adverse prognostic pattern for higher PIV after PCI, although the magnitude, consistency, and certainty of evidence differed substantially across outcome domains, with moderate certainty for long-term all-cause mortality and angiographic no-reflow or slow-flow, low certainty for composite MACE and cardiac mortality, and very low certainty for post-contrast renal injury under the GRADE framework.

For the primary outcome of study-defined major adverse cardiovascular events (MACE), higher PIV was associated with an increased risk on the adjusted hazard scale (pooled adjusted HR 1.65, 95% CI 1.20–2.27; k = 2; I² = 0.0%) and showed moderate but heterogeneous discrimination on the AUC scale (pooled AUC 0.729, 95% CI 0.578–0.841; k = 5; I² = 83.0%). The overall certainty of evidence for MACE was rated as low, downgraded for risk of bias and for inconsistency in the discrimination evidence.

Among secondary outcomes, the most consistent association was observed for long-term all-cause mortality, where higher PIV was associated with an approximately 3.5-fold higher adjusted hazard of death (pooled adjusted HR 3.51, 95% CI 2.15–5.74; k = 2; I² = 0.0%; moderate certainty), with directionally and quantitatively concordant estimates from the two contributing studies. A single component-level estimate from a non-ST-elevation ACS cohort additionally suggested an association with cardiac mortality (adjusted HR 3.24, 95% CI 1.34–7.81; n = 1,606; events = 24; low certainty). For angiographic no-reflow or slow-flow, PIV demonstrated good and consistent discriminatory performance across two retrospective ST-elevation myocardial infarction primary-PCI cohorts (pooled AUC 0.828, 95% CI 0.808–0.846; k = 2; I² = 0.0%; moderate certainty).

The weakest and most heterogeneous evidence pertained to post-contrast renal injury outcomes. Although the pooled AUC for post-contrast renal injury was moderate on average (0.771, 95% CI 0.617–0.875; k = 5), between-study heterogeneity was very high (I² = 95.1%), and an exploratory pooling of high-versus-low adjusted odds ratios yielded a wide confidence interval that crossed the null (OR 4.91, 95% CI 0.87–27.89; k = 2; very low certainty). Taken together, these findings indicate that PIV appears to carry a consistent prognostic signal for long-term mortality and a consistent discriminatory signal for angiographic no-reflow or slow-flow in ACS patients undergoing PCI, while its association with composite cardiovascular events is detectable but supported by sparse adjusted association data, and its discriminatory performance for renal outcomes is highly variable. These observations should be interpreted within the constraints of the available evidence, including observational study designs, small numbers of studies for several outcomes, and the geographic concentration of the included cohorts, which together support a cautious framing of PIV as a candidate supplementary biomarker requiring further validation rather than as an established clinical marker.

A growing body of evidence has supported the use of peripheral blood-derived inflammatory indices as candidate prognostic biomarkers in patients with ACS undergoing PCI. In the ACS-PCI literature, four related indices have received particularly extensive investigation: the neutrophil-to-lymphocyte ratio (NLR), the platelet-to-lymphocyte ratio (PLR), the systemic immune-inflammation index (SII; calculated as platelet × neutrophil/lymphocyte), and the systemic inflammation response index (SIRI; calculated as neutrophil × monocyte/lymphocyte). Prior meta-analyses have linked these indices to adverse outcomes including all-cause mortality, MACE, angiographic no-reflow, and post-contrast renal injury (8–10, 43). PIV differs from these earlier indices principally in its compositional structure: whereas NLR and PLR integrate two leukocyte or platelet–leukocyte populations and SII and SIRI integrate three, PIV simultaneously incorporates four cellular components—neutrophils, platelets, monocytes, and lymphocytes—within a single composite expression. This compositional difference describes the structural arrangement of PIV relative to earlier indices but does not in itself establish superior clinical performance.

**Figure 5:**
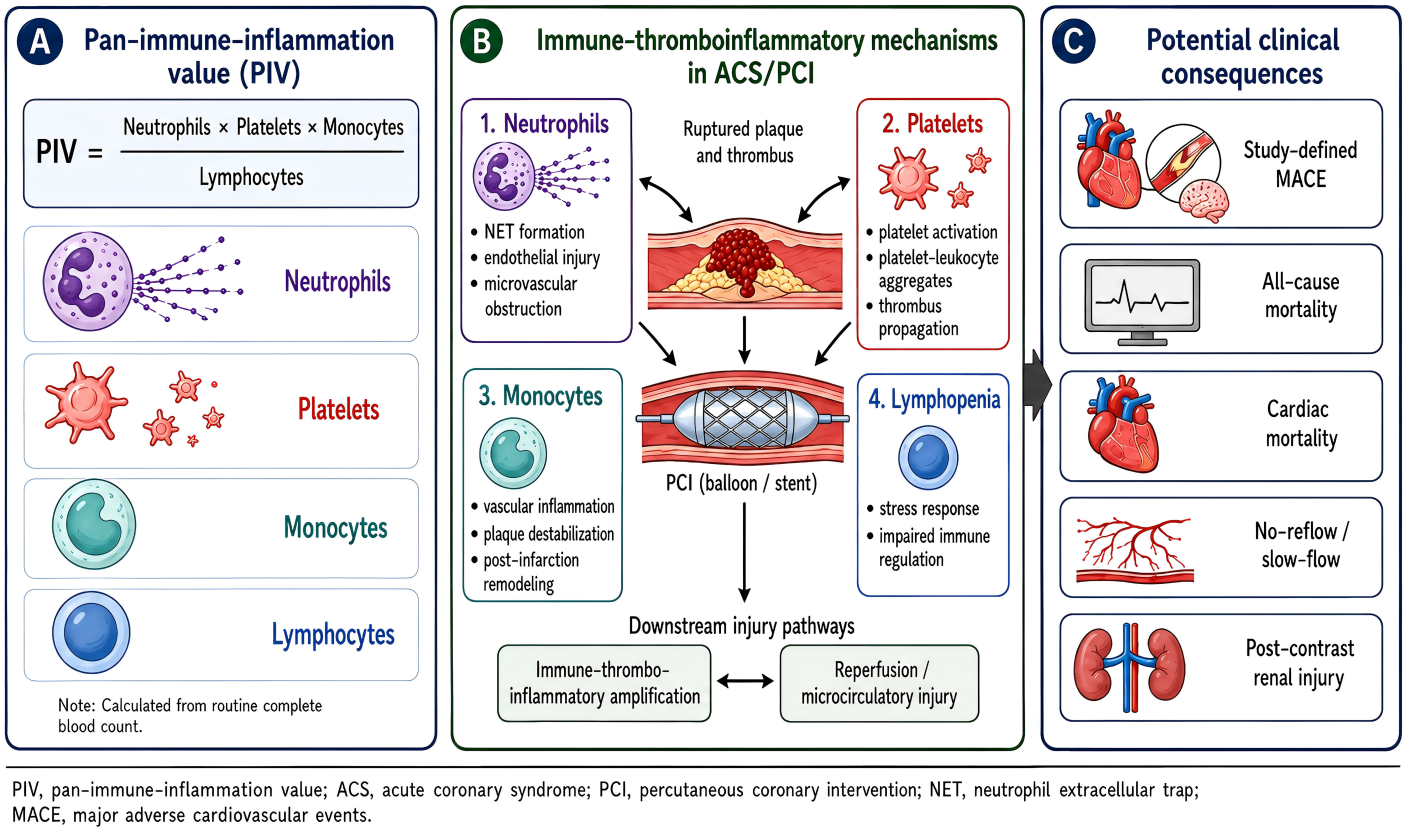
Pan-immune-inflammation value and its proposed immune-thromboinflammatory framework in acute coronary syndrome and percutaneous coronary intervention. **(A)** PIV is a composite peripheral blood index calculated from absolute neutrophil, platelet, monocyte, and lymphocyte counts on routine complete blood count testing. **(B)** The four cellular components map onto interrelated mechanistic axes: neutrophil-driven NET formation and microvascular obstruction, platelet activation and thromboinflammation, monocyte-mediated vascular inflammation and remodelling, and lymphopenia reflecting stress response and impaired immune regulation. These axes may converge on immune-thromboinflammatory amplification and reperfusion-related microcirculatory injury during and after PCI. **(C)** These mechanisms may contribute to the adverse cardiovascular and renal outcomes evaluated in the present synthesis. ACS, acute coronary syndrome; NET, neutrophil extracellular trap; PCI, percutaneous coronary intervention; PIV, pan-immune-inflammation value.

This four-component structure provides a conceptual rationale for PIV as an integrative immune-thromboinflammatory index. Neutrophils reflect innate immune activation, platelets capture thromboinflammatory and procoagulant activity, monocytes represent vascular inflammatory and reparative responses, and lymphocyte counts reflect adaptive immune status and systemic stress responses (5, 6, 44). By integrating all four cellular axes within a single expression, PIV may capture a wider spectrum of immune-inflammatory perturbation than indices restricted to two or three of these components. This conceptual advantage, however, remains a theoretical proposition and requires direct empirical confirmation. The biological pathways linking these four cellular components to adverse cardiovascular and renal outcomes after ACS and PCI are summarised in **Figure 5** and discussed below.

Despite this conceptual rationale, the present meta-analysis does not establish whether PIV is superior, equivalent, or inferior to NLR, PLR, SII, or SIRI for prognostication in this population. Several included studies reported within-study comparisons of receiver operating characteristic curves or regression estimates across multiple inflammatory indices, but these comparisons were neither uniformly available nor methodologically standardised across studies, and were not amenable to pooled synthesis (12–14, 17, 42). PIV should therefore currently be regarded as a biologically plausible supplementary marker alongside established inflammatory biomarkers, rather than a replacement for them, in ACS-PCI prognostication. Whether PIV provides incremental discrimination, calibration, or net reclassification improvement over NLR, PLR, SII, or SIRI remains unproven. Adequately powered prospective studies that prespecify direct comparisons of multiple indices within the same cohort and against the same reference outcomes will be needed to clarify the relative position of PIV among these biomarkers.

The four cellular components of PIV map onto four interrelated axes of post-PCI cardiovascular injury, which may converge through immune-thromboinflammatory pathways and contribute to microvascular obstruction, ischaemia–reperfusion injury, post-contrast renal injury, and longer-term adverse cardiovascular outcomes (4–6, 45, 46). **Figure 5** summarises the proposed integrative framework linking the cellular components of PIV to the clinical outcomes evaluated in the present synthesis.

Neutrophils represent a principal effector arm of acute innate immune activation in ACS and may contribute to post-PCI injury through several non-mutually exclusive mechanisms, among which neutrophil extracellular trap (NET) formation is among the best-characterised (5). NETs are released by activated neutrophils at sites of plaque rupture, promote microvascular thrombus formation, and may physically obstruct distal capillaries, thereby contributing to angiographic no-reflow and slow-flow after primary PCI (3, 47). They may also promote endothelial injury and amplify local cytokine release, extending tissue damage beyond the initial ischaemic insult. The consistent discriminatory performance of PIV for no-reflow in the present synthesis is compatible with prior evidence implicating neutrophil-driven microvascular obstruction in this outcome.

Platelets contribute the thromboinflammatory axis. Beyond their canonical role in haemostasis, activated platelets form physical aggregates with circulating leukocytes—particularly platelet–neutrophil and platelet–monocyte aggregates—through P-selectin/PSGL-1 and CD40L-mediated interactions (45). These heterotypic aggregates can amplify vascular inflammation, promote NET formation, and enhance local procoagulant activity. Within the framework of immunothrombosis, platelet activation and innate immune activation are mechanistically coupled rather than parallel processes, providing a rationale for integrating both platelet and leukocyte counts within a single biomarker (48, 49).

Monocytes mediate vascular inflammation, plaque destabilisation, and post-infarction myocardial remodelling. Classical and intermediate monocyte subsets are recruited after myocardial infarction and have been associated with larger infarct size, impaired myocardial salvage, and adverse left ventricular remodelling (6, 50). The inclusion of monocytes within PIV—a feature shared with SIRI but not with NLR, PLR, or SII—may therefore be relevant for outcomes reflecting cumulative myocardial damage and remodelling, including longer-term mortality and composite MACE.

Lymphocyte counts capture a fourth, partially distinct dimension. Stress-induced lymphopenia in ACS reflects catecholamine- and cortisol-driven redistribution and is a recognised marker of physiological stress severity; concurrently, reduced lymphocyte counts may reflect impaired adaptive immune regulation, which can contribute to maladaptive post-infarction inflammation (7, 44). By placing lymphocyte counts in the denominator of PIV, the index increases in patients with simultaneous innate immune activation, platelet-related thromboinflammatory activity, monocytic inflammation, and relative lymphopenia.

The clinical convergence of these four axes may also be relevant to post-contrast renal injury, which arises from a multifactorial cascade involving tubular toxicity, medullary hypoxia, oxidative stress, endothelial dysfunction, and neutrophil-mediated microvascular inflammation (51). Long-term mortality after ACS reflects the cumulative burden of microvascular injury, infarct size, adverse remodelling, and recurrent thromboinflammatory events, again paralleling the biological construct that PIV is intended to approximate (4, 52). This mechanistic framework provides a coherent biological rationale for the prognostic and discriminatory signals observed in the present meta-analysis, while also highlighting the limits of inferring mechanism from cell-count-derived indices alone—a caveat that should be considered when interpreting the limitations of the current evidence and future research priorities.

PIV is derived from a routine complete blood count and requires no additional sampling, processing, or laboratory infrastructure beyond what is already available for most ACS patients undergoing PCI in contemporary hospital settings. This operational simplicity, shared with NLR, PLR, SII, and SIRI, makes PIV attractive as a candidate addition to risk stratification in this population (8, 11). The findings of the present synthesis suggest that baseline PIV, measured at admission or before PCI, may help identify patients with higher long-term mortality risk and may provide discriminatory information for angiographic no-reflow or slow-flow, subject to further validation, with more cautious interpretation required for composite MACE, cardiac mortality, and post-contrast renal injury.

Despite this potential, several considerations currently constrain the direct translation of PIV into routine clinical practice. The most important of these is the substantial heterogeneity in cut-off values across the included studies, with ROC-derived thresholds varying more than fourfold, from approximately 223 to 906. This dispersion likely reflects differences in study populations, ACS subtypes, target outcomes, and methods of cut-off derivation, and precludes the recommendation of a single threshold for clinical decision-making at the present time, particularly across different outcome domains. Future prospective work will be needed to establish externally validated, outcome- and population-specific PIV thresholds before the index can support categorical risk classification in routine care.

Within these constraints, PIV is best positioned at this stage as a supplementary marker that complements rather than replaces established risk stratification tools such as the GRACE score, the TIMI risk score, and conventional cardiovascular risk factor assessment (53, 54). Whether PIV provides incremental discrimination, calibration, or net reclassification improvement over and above these established scores remains unproven and could not be formally synthesised in the present meta-analysis; this represents an important question for future incremental-value studies. Dynamic assessment of PIV may offer additional prognostic information, but evidence on serial PIV trajectories remains preliminary and was not formally synthesised.

This systematic review and meta-analysis has several methodological strengths. The eligibility criteria, search strategy, statistical synthesis plan, and risk-of-bias and certainty assessment frameworks were prespecified and prospectively registered with PROSPERO before data extraction commenced (CRD420261378751). The search covered six electronic databases, including PubMed/MEDLINE, Embase, Web of Science Core Collection, the Cochrane Library/CENTRAL, Scopus, and CNKI, and was supplemented by trial registry searches, screening of the first 200 Google Scholar results, and hand-searching of reference lists, with no language restrictions applied. Study selection, data extraction, QUIPS risk-of-bias assessment, and GRADE certainty rating were performed independently by two reviewers, with disagreements resolved through discussion and consensus. The synthesis strategy maintained strict separation between effect-measure types: adjusted HRs, ORs, and AUC values were pooled separately and were not combined within the same analysis. A consistent random-effects framework was applied across outcomes, and feasible sensitivity analyses, including leave-one-out analyses and fixed-effect versus random-effects comparisons, were conducted where data permitted.

Several important limitations qualify these strengths and should be considered when interpreting the findings. First, all included studies were observational, and residual confounding from unmeasured procedural, pharmacological, or patient-level variables cannot be excluded, even among studies reporting multivariable-adjusted estimates. Risk-of-bias concerns were also common: no study was judged to have low overall risk of bias by the QUIPS tool, and three studies—Keskin et al. (32), Ömür et al. (38), and Zorlu et al. (41)—were rated at high overall risk of bias because of insufficient PIV-specific multivariable adjustment or limitations in statistical reporting. Second, the number of studies contributing to each outcome was small (k = 1 to 5), which precluded formal assessment of publication bias using funnel plots or Egger’s regression test, as well as formal subgroup analysis and meta-regression. The limited number of studies also meant that several clinically relevant factors, including ACS subtype, follow-up duration, PIV measurement timing, and cut-off derivation method, could only be considered narratively. Third, the evidence base was geographically concentrated: 17 of 19 studies were conducted in Türkiye or mainland China, leaving the generalisability of the findings to Western European, North American, and other populations uncertain. Fourth, clinical and methodological heterogeneity was substantial. PIV measurement timing varied across admission, pre-PCI, within 12 hours of admission, and within 24 hours of symptom onset; MACE definitions were retained as study-defined and differed in their components; post-contrast renal injury was reported under multiple labels, including CA-AKI, CI-AKI, CIN, and PCAKI; and ROC-derived PIV cut-offs varied more than fourfold across studies. These sources of heterogeneity limit the interpretability of pooled estimates and preclude the recommendation of a single clinical PIV threshold. Finally, the available evidence did not allow formal synthesis of the incremental value of PIV over established risk scores such as the GRACE score or the TIMI risk score, nor could dynamic PIV trajectories during hospitalisation or early follow-up be synthesised. Taken together, these limitations support a cautious interpretation of the present findings and reinforce the need for prospective, externally validated studies using standardised outcome definitions, prespecified PIV measurement windows, and direct comparisons with established inflammatory indices and clinical risk scores.

The present synthesis highlights several priorities for future research. First, prospective multicentre cohort studies enrolling ACS patients undergoing PCI in Western European, North American, and other under-represented populations are needed to test the external validity of the present findings, given the strong concentration of existing evidence in Türkiye and mainland China. Second, head-to-head comparative studies that prespecify the simultaneous evaluation of PIV alongside NLR, PLR, SII, and SIRI within the same cohorts and against the same reference outcomes are needed to clarify the incremental value and relative prognostic and discriminatory position of PIV among related inflammatory indices. Third, methodological standardisation of PIV measurement timing—particularly whether admission or pre-PCI sampling should serve as the reference point, and how post-PCI or follow-up measurements should be analysed separately—together with outcome- and population-specific cut-off derivation would improve the comparability and clinical interpretability of future evidence. Fourth, the incremental value of PIV over established risk stratification tools, including the GRACE score and the TIMI risk score, should be formally evaluated using changes in discrimination, calibration, net reclassification improvement, integrated discrimination improvement, and decision-curve analysis. Fifth, dynamic assessment of PIV through serial measurements during the index hospitalisation and early follow-up may capture temporal changes in immune-thromboinflammatory activation and identify trajectory phenotypes with distinct prognostic implications. Finally, mechanistic clinical studies linking PIV to imaging-based markers of microvascular obstruction and infarct size on cardiac magnetic resonance imaging, as well as circulating markers of NET formation and platelet–leukocyte interactions, would strengthen the biological interpretation of PIV beyond its derivation from routine cell counts.

## Conclusion

In ACS patients undergoing PCI, elevated pan-immune-inflammation value was associated with study-defined MACE and other adverse cardiovascular outcomes. The strongest and most consistent evidence supported its association with long-term all-cause mortality and its discriminatory performance for angiographic no-reflow or slow-flow; these two outcome domains were supported by moderate-certainty evidence. Associations with composite MACE were directionally consistent but supported by sparse adjusted association data and heterogeneous discrimination performance (low certainty), whereas evidence for cardiac mortality (low certainty) and post-contrast renal injury (very low certainty) remained weaker and more heterogeneous. Considerable variability in cut-off values, measurement timing, outcome definitions, and risk-of-bias profiles across the available literature, together with marked geographic concentration of existing studies, currently precludes the recommendation of a single PIV threshold for clinical use. PIV is best regarded at this stage as a biologically plausible supplementary marker that complements established risk stratification tools**, and its incremental value over these established tools remains unproven**. Adequately powered prospective multicentre studies and head-to-head comparisons with established inflammatory indices and clinical risk scores are needed before its clinical role can be defined.

## Data Availability

The original contributions presented in the study are included in the article/**Supplementary Material**. Further inquiries can be directed to the corresponding author.
