# Supplementary material for: Pan-immune-inflammation value for risk stratification of adverse cardiovascular outcomes in acute coronary syndrome undergoing percutaneous coronary intervention: a systematic review and meta-analysis
Source: Front Immunol. 2026 Jun 1;17:1870678. doi: 10.3389/fimmu.2026.1870678 (PMC13266294; doi:10.3389/fimmu.2026.1870678)
Supplement: Supplementary file 1 [file DataSheet1.pdf]

Supplementary Table S1. Database-specific search strategies

Search concept structure: PIV terms AND ACS terms AND PCI terms

Search date: 2026-04-25

Language restrictions: None

Date restrictions: From database inception to 2026-04-25

Database 1. PubMed/MEDLINE

Interface: PubMed (https://pubmed.ncbi.nlm.nih.gov) **Records retrieved:** 22 | **Search date:** 2026-04-25

#	**Query**
#1	"pan-immune-inflammation value"[Title/Abstract] OR "pan immune inflammation value"[Title/Abstract] OR "panimmune inflammation value"[Title/Abstract] OR "pan-immune inflammation value"[Title/Abstract] OR PIV[Title/Abstract]
#2	"Acute Coronary Syndrome"[MeSH Terms] OR "Myocardial Infarction"[MeSH Terms] OR "acute coronary syndrome"[Title/Abstract] OR "acute coronary syndromes"[Title/Abstract] OR ACS[Title/Abstract] OR "myocardial infarction"[Title/Abstract] OR "ST-elevation myocardial infarction"[Title/Abstract] OR STEMI[Title/Abstract] OR "non-ST-elevation myocardial infarction"[Title/Abstract] OR NSTEMI[Title/Abstract] OR "non ST elevation myocardial infarction"[Title/Abstract] OR "unstable angina"[Title/Abstract]
#3	"Percutaneous Coronary Intervention"[MeSH Terms] OR "percutaneous coronary intervention"[Title/Abstract] OR "percutaneous coronary interventions"[Title/Abstract] OR PCI[Title/Abstract] OR "coronary intervention"[Title/Abstract] OR "coronary interventions"[Title/Abstract] OR "coronary stent"[Title/Abstract] OR "coronary stenting"[Title/Abstract] OR angioplasty[Title/Abstract]
**#4**	**#1 AND #2 AND #3**

Database 2. Embase

Interface: Embase.com (Elsevier) **Records retrieved:** 31 | **Search date:** 2026-04-25

#	Query
#1	'pan-immune-inflammation value':ti,ab,kw OR 'pan immune inflammation value':ti,ab,kw OR 'panimmune inflammation value':ti,ab,kw OR 'pan-immune inflammation value':ti,ab,kw OR piv:ti,ab,kw
#2	'acute coronary syndrome'/exp OR 'heart infarction'/exp OR 'unstable angina pectoris'/exp OR 'acute coronary syndrome':ti,ab,kw OR 'acute coronary syndromes':ti,ab,kw OR acs:ti,ab,kw OR 'myocardial infarction':ti,ab,kw OR 'st elevation myocardial infarction':ti,ab,kw OR 'st-elevation myocardial infarction':ti,ab,kw OR stemi:ti,ab,kw OR 'non st elevation myocardial infarction':ti,ab,kw OR 'non-st-elevation myocardial infarction':ti,ab,kw OR nstemi:ti,ab,kw OR 'unstable angina':ti,ab,kw
#3	'percutaneous coronary intervention'/exp OR 'coronary artery stent'/exp OR 'percutaneous transluminal coronary angioplasty'/exp OR 'percutaneous coronary intervention':ti,ab,kw OR 'percutaneous coronary interventions':ti,ab,kw OR pci:ti,ab,kw OR 'coronary intervention':ti,ab,kw OR 'coronary interventions':ti,ab,kw OR 'coronary stent':ti,ab,kw OR 'coronary stenting':ti,ab,kw OR angioplasty:ti,ab,kw
**#4**	**#1 AND #2 AND #3**

Database 3. Web of Science Core Collection

Interface: Web of Science (Clarivate) **Indexes searched:** SCI-EXPANDED, SSCI, ESCI **Records retrieved:** 22 | **Search date:** 2026-04-25

#	Query
#1	TS=("pan-immune-inflammation value" OR "pan immune inflammation value" OR "panimmune inflammation value" OR "pan-immune inflammation value" OR PIV)
#2	TS=("acute coronary syndrome" OR "acute coronary syndromes" OR ACS OR "myocardial infarction" OR "ST-elevation myocardial infarction" OR "ST elevation myocardial infarction" OR STEMI OR "non-ST-elevation myocardial infarction" OR "non ST elevation myocardial infarction" OR NSTEMI OR "unstable angina")
#3	TS=("percutaneous coronary intervention" OR "percutaneous coronary interventions" OR PCI OR "coronary intervention" OR "coronary interventions" OR "coronary stent" OR "coronary stenting" OR angioplasty)
**#4**	**#1 AND #2 AND #3**

Database 4. Scopus

Interface: Scopus (Elsevier) **Records retrieved:** 24 | **Search date:** 2026-04-25

#	Query
#1	TITLE-ABS-KEY ("pan-immune-inflammation value" OR "pan immune inflammation value" OR "panimmune inflammation value" OR "pan-immune inflammation value" OR PIV)
#2	TITLE-ABS-KEY ("acute coronary syndrome" OR "acute coronary syndromes" OR ACS OR "myocardial infarction" OR "ST-elevation myocardial infarction" OR "ST elevation myocardial infarction" OR STEMI OR "non-ST-elevation myocardial infarction" OR "non ST elevation myocardial infarction" OR NSTEMI OR "unstable angina")
#3	TITLE-ABS-KEY ("percutaneous coronary intervention" OR "percutaneous coronary interventions" OR PCI OR "coronary intervention" OR "coronary interventions" OR

#	Query
	"coronary stent" OR "coronary stenting" OR angioplasty)
**#4**	**#1 AND #2 AND #3**

Database 5. Cochrane Library (CENTRAL)

Interface: Cochrane Library Advanced Search (https://www.cochranelibrary.com) **Records retrieved:** 1 | **Search date:** 2026-04-25

#	Query
#1	"pan-immune-inflammation value" OR "pan immune inflammation value" OR "panimmune inflammation value" OR "pan-immune inflammation value" OR PIV
#2	"acute coronary syndrome" OR "acute coronary syndromes" OR ACS OR "myocardial infarction" OR "ST-elevation myocardial infarction" OR "ST elevation myocardial infarction" OR STEMI OR "non-ST-elevation myocardial infarction" OR "non ST elevation myocardial infarction" OR NSTEMI OR "unstable angina"
#3	"percutaneous coronary intervention" OR "percutaneous coronary interventions" OR PCI OR "coronary intervention" OR "coronary interventions" OR "coronary stent" OR "coronary stenting" OR angioplasty
**#4**	**#1 AND #2 AND #3**

Database 6. China National Knowledge Infrastructure (CNKI)

Interface: CNKI (https://www.cnki.net) **Search field:** Subject (主题) **Records retrieved:** 0 | **Search date:** 2026-04-25

#	Query
#1	"泛免疫炎症值" OR "泛免疫炎症指数" OR "泛免疫炎症评分" OR "全免疫炎症值" OR "全免疫炎症指数" OR "pan-immune-inflammation value" OR "pan immune inflammation value" OR "PIV"
#2	"急性冠脉综合征" OR "急性冠状动脉综合征" OR "急性心肌梗死" OR "ST 段抬高型心肌梗死" OR "非 ST 段抬高型心肌梗死" OR "不稳定型心绞痛" OR "ACS" OR "STEMI" OR "NSTEMI"
#3	"经皮冠状动脉介入" OR "经皮冠脉介入" OR "经皮冠状动脉介入治疗" OR "经皮冠脉介入治疗" OR "冠状动脉介入" OR "冠脉介入" OR "PCI" OR "支架" OR "冠状动脉支架"
**#4**	**#1 AND #2 AND #3**

Database 7. ClinicalTrials.gov

Interface: ClinicalTrials.gov (https://clinicaltrials.gov) **Records retrieved:** 0 (across all 5 sequential queries) | **Search date:** 2026-04-25

Owing to the platform's limited support for nested Boolean syntax, the following queries were executed sequentially and the resulting records were combined and deduplicated:

#	Query	Records retrieved
Q1	"pan-immune-inflammation value" AND "acute coronary syndrome" AND "percutaneous coronary intervention"	0
Q2	PIV AND "acute coronary syndrome" AND PCI	0
Q3	"pan immune inflammation value" AND myocardial infarction	0
Q4	PIV AND STEMI	0
Q5	PIV AND NSTEMI	0
**Total after deduplication**		**0**

Additional sources screened

The following sources were also searched to identify ongoing or unpublished studies and to capture potentially eligible records not indexed in the primary databases:

- **WHO International Clinical Trials Registry Platform (ICTRP)** — searched on 2026-04-25 using core terms: *pan-immune-inflammation value*, *PIV*, *acute coronary syndrome*, *percutaneous coronary intervention*. No additional eligible records identified.
- **Google Scholar** — first 200 records sorted by relevance, screened on 2026-04-25 using the query: *"pan-immune-inflammation value" AND "acute coronary syndrome" AND "percutaneous coronary intervention"*. No additional eligible records identified.
- **Reference lists** of all included studies and relevant reviews — hand-searched for additional eligible records. No additional eligible records identified.

Notes on search strategy

Concept blocks: The search combined three concept blocks — (i) pan-immune-inflammation value (the prognostic factor of interest), (ii) acute coronary syndrome (the population), and (iii) percutaneous coronary intervention (the clinical context). No outcome-related terms were included in the search to maximise sensitivity, as outcome data were frequently reported only in the full text or results tables of primary studies.

Controlled vocabulary: Where supported by the database, controlled vocabulary terms (MeSH for PubMed, Emtree for Embase) were combined with free-text terms in title, abstract, and keyword fields.

PIV abbreviation: The abbreviation *PIV* was retained in all searches. While *PIV* is not unique to pan-immune-inflammation value, the requirement for simultaneous matching of ACS and PCI terms substantially reduced irrelevant retrieval.

Chinese-language search: CNKI was searched using both Chinese terminology and English equivalents, as Chinese-language journals frequently include English titles, abstracts, and keywords. Multiple Chinese variants of *pan-immune-inflammation value* were included to ensure comprehensive retrieval.

Adaptation across databases: The core logic was preserved across all databases. Database-specific syntax (field tags, proximity operators, controlled vocabulary syntax) was adapted as required by each interface. Substantive concept terms remained consistent.

Search yield summary

Source	Records retrieved
PubMed/MEDLINE	22
Embase	31
Web of Science Core Collection	22
Scopus	24
Cochrane Library/CENTRAL	1
CNKI	0
ClinicalTrials.gov	0
WHO ICTRP	0
Google Scholar (first 200)	0 additional
Hand-searching reference lists	0 additional
**Total before deduplication**	**100**
**After removal of 68 duplicate records**	**32 unique records**

Supplementary Table S2A. Full-text articles excluded with reasons (n = 2)

First author, year	Title	Journal	DOI/PMID	Reason for exclusion (PECOS domain)
Duan et al., 2026	The Pan-Immune-Inflammation Value (PIV) predicts major adverse cardiovascular events in elderly patients undergoing percutaneous coronary intervention: a real-world study.	BMC Cardiovascular Disorders	DOI: 10.1186/s12872-026-05679-y; PMID: 41781875	P (Population): elderly PCI cohort not restricted to adults with index acute coronary syndrome undergoing PCI; ACS-PCI subgroup data were not separately extractable.
Karaduman et al., 2024	Prognostic value of pan immune-inflammation value in patients undergoing unprotected left main coronary artery stenting.	Biomarkers in Medicine	DOI: 10.1080/17520363.2024.2412515	P (Population): unprotected left main coronary artery PCI cohort included stable coronary artery disease/chronic coronary syndrome as well as ACS presentations; ACS-PCI subgroup data were not separately extractable.

Full-text articles were excluded after eligibility assessment according to the predefined PECOS criteria specified in the registered review protocol (PROSPERO CRD420261378751). These full-text exclusions are distinct from included studies retained for qualitative synthesis only. Studies with available full text that met PECOS criteria but did not provide a compatible PIV-specific adjusted effect estimate or extractable AUC were retained in the systematic review and are not listed here. In the final synthesis, 19 studies were included in the review; 14 contributed to at least one pooled quantitative estimate and five were retained for qualitative synthesis only (Keskin et al., 2026; Ma et al., 2025; Genc et al., 2025; Yang et al., 2024; Murat et al., 2023).

Abbreviations: ACS, acute coronary syndrome; AUC, area under the receiver operating characteristic curve; PCI, percutaneous coronary intervention; PECOS, Population, Exposure, Comparator, Outcomes, Study design; PIV, pan-immune-inflammation value.

Supplementary Table S2B. Reports sought for retrieval but not retrieved (n = 2)

First author, year	Title	Journal / Source	Publication type	DOI / PMID / Other identifier	Sources where indexed	Retrieval attempts and outcomes
Cetinkaya et al., 2025	The Role of Pan-Immune-Inflammation Value in Predicting Contrast-Induced Nephropathy Development in Patients Undergoing Percutaneous Coronary Intervention Due to NSTEMI.	Angiology. 2025;76(3):281-288.	Journal article	DOI: 10.1177/00033197231211107; PMID: 37903550	PubMed/MEDLINE; Embase; Web of Science Core Collection; Scopus	Retrieval attempts: (i) Peking University library system — full text not accessible (ii) Publisher journal website — paywall, access denied (iii) Corresponding author email not retrievable from the article or indexing databases
Çetinkaya, 2023	The role of new inflammatory markers in determining the development of contrast-induced nephropathy in patients undergoing percutaneous coronary angiography due to STEMI.	Anatolian Journal of Cardiology. 2023;27(Suppl):S213-S214.	Conference abstract	Embase accession ID: L643265276	Embase only	(i) PubMed/MEDLINE, Web of Science Core Collection, Scopus, and web searches were checked for a subsequent full-length publication by title and author: no full-length report identified; (ii) Anatolian Journal of Cardiology conference supplement information indicated an abstract-only record; (iii) corresponding-author contact: not documented as completed at the time this table was prepared.

Reports judged potentially eligible at title and abstract screening for which the full text or full-length report could not be obtained despite documented retrieval attempts, including institutional library access, publisher/journal websites, and searches for subsequent full-length publications where applicable. These reports could neither be included nor formally excluded under the predefined PECOS eligibility criteria specified in the registered review protocol (PROSPERO CRD420261378751) due to the absence of full text or full-length report. Reports not retrieved are distinct from included qualitative-only studies; the latter had full text available, met PECOS criteria, and were retained in the systematic review but not pooled quantitatively because no compatible PIV-specific adjusted effect estimate or extractable AUC was available. In the final synthesis, 19 studies were included in the review; 14 contributed to at least one pooled quantitative estimate and five were retained for qualitative synthesis only (Keskin et al., 2026; Ma et al., 2025; Genc et al., 2025; Yang et al., 2024; Murat et al., 2023).
Abbreviations: ACS, acute coronary syndrome; AUC, area under the receiver operating characteristic curve; NSTEMI, non-ST-segment elevation myocardial infarction; PCI, percutaneous coronary intervention; PECOS, Population, Exposure, Comparator, Outcomes, Study design; PIV, pan-immune-inflammation value; PMID, PubMed identifier; STEMI, ST-segment elevation myocardial infarction.

Supplementary Table S3. Study-specific definitions and components of major adverse cardiovascular events (MACE) across included studies.

First author, year	Country	Composite outcome name as reported	Component 1	Component 2	Component 3	Component 4	Component 5+	Follow-up duration	Notes
Çiçek et al., 2026	Türkiye	Not reported (single outcome: contrast-associated acute kidney injury)	Contrast-associated acute kidney injury	-	-	-	-	48-72 h after PCI	CA-AKI defined as >25% or >=0.5 mg/dL increase in serum creatinine within 48-72 h after the procedure (KDIGO-equivalent criteria); admission PIV evaluated in STEMI treated with primary PCI.
Keskin et al., 2026	Türkiye	Not reported (single outcome: long-term mortality)	All-cause mortality	-	-	-	-	24 months	Machine-learning mortality model; PIV included as a candidate inflammatory-metabolic index, but no composite MACE outcome was defined.
Qu et al., 2026	China	Major adverse cardiovascular events (MACE)	Recurrent acute myocardial infarction	Malignant arrhythmias	Heart failure	Cardiogenic shock	All-cause mortality	12-month follow-up after PCI	Components described as 'including but not limited to'; the original publication did not specify whether outcomes were adjudicated by an independent committee blinded to PIV.
Ma et al., 2025	China	Not reported (single outcome: in-hospital heart failure)	In-hospital heart failure	-	-	-	-	In-hospital only	Prospective elderly AMI cohort after successful PCI; patients with baseline heart failure were excluded.
Chen et al., 2025	China	Major adverse cardiovascular	All-cause mortality	Non-fatal reinfarction /	Target vessel revascularizat	Cerebrovascular events	Hospital readmissions	Median 12 months	MI subgroup after UAP diagnosis

First author, year	Country	Composite outcome name as reported	Component 1	Component 2	Component 3	Component 4	Component 5+	Follow-up duration	Notes
		events (MACE)		recurrent MI	ion		for heart failure		underwent PCI and was followed for post-PCI MACE.
Konte et al., 2025	Türkiye	Not reported (single outcome: contrast-induced acute kidney injury)	Contrast-induced acute kidney injury	-	-	-	-	48-72 h after contrast exposure	CI-AKI defined as >=0.5 mg/dL absolute or >=25% relative increase in serum creatinine within 48-72 h after contrast exposure.
Xu et al., 2025	China	Major adverse cardiovascular events (MACE)	Malignant arrhythmias	Severe heart failure	Recurrent nonfatal myocardial infarction	All-cause mortality	-	6 months	Endpoint events reviewed and confirmed by an independent Clinical Events Committee blinded to biomarker data; PIV assessed dynamically within the first 24 h after onset.
Ting et al., 2025	Taiwan	Not reported (primary single outcome: 1-year all-cause mortality)	All-cause mortality	Reinfarction (secondary outcome)	-	-	-	Primary/secondary endpoints assessed at 1 year; median overall follow-up 4.7 years	Mortality ascertained through Taiwan National Cause of Death Registry; reinfarction identified within 1 year.
Liu et al., 2025	China	Major adverse cardiovascular events (MACEs)	Cardiogenic mortality	Rehospitalization for severe heart failure	Recurrence of myocardial infarction	In-stent restenosis	-	Median 1,006 days (~33 months); follow-up visits at 1, 3, 6, and 12 months, and annually thereafter	Severe heart failure defined as NYHA class IV; long-term event-free survival endpoint.
Byoun et al., 2025	South Korea	Major adverse cardiovascular events (MACEs)	All-cause death	Myocardial infarction	Stroke	Any revascularization	Rehospitalization for heart failure	1 year; median 400 days (IQR 367.8-400)	First event counted if multiple components occurred; individual

First author, year	Country	Composite outcome name as reported	Component 1	Component 2	Component 3	Component 4	Component 5+	Follow-up duration	Notes
									components also reported.
Ömür et al., 2024	Türkiye	Not reported (single outcome: contrast-induced nephropathy)	Contrast-induced nephropathy	-	-	-	-	48-72 h / within 3 days after contrast exposure	Retrospective ACS cohort (USAP, NSTEMI, STEMI); PIV correlated with Mehran and SYNTAX scores among patients developing CIN.
Genc et al., 2025	Türkiye	Not reported (single outcome: in-hospital all-cause mortality)	In-hospital all-cause mortality	-	-	-	-	In-hospital only	Derivation and validation cohorts; PIV compared with modified Naples Prognostic Score (mNPS) and other inflammatory indices.
Yang et al., 2024	China	MACE during hospitalization	All-cause death	New heart failure after AMI	Recurrent infarction	Life-threatening arrhythmias	-	In-hospital events; all-cause death assessed during hospitalization with 1-week follow-up per original definition	Inpatient MACE was the analytic endpoint; severe coronary stenosis was a separate outcome.
Kurtul and Gok, 2024	Türkiye	Not reported (single outcome: postcontrast acute kidney injury)	Postcontrast acute kidney injury	-	-	-	-	Within 72 h after PCI	PCAKI defined as >=0.5 mg/dL and/or >=25% serum creatinine increase within 72 h after PCI; ACS patients treated with DES.
Şen et al., 2024	Türkiye	Not reported (single outcome: impaired coronary flow)	Impaired coronary flow / no-reflow	-	-	-	-	Immediate post-pPCI angiographic assessment	ICF defined as postprocedural TIMI 0-2 or TIMI 3 with myocardial blush grade <2; final flow checked

First author, year	Country	Composite outcome name as reported	Component 1	Component 2	Component 3	Component 4	Component 5+	Follow-up duration	Notes
									by blinded interventional cardiologist.
Liu et al., 2023	China	Major adverse cardiac/cardiovascular events (MACEs)	All-cause death	Recurrent MI / acute reinfarction	Stent reimplantation for unstable angina	Heart failure	Combined events (reinfarction + heart failure; revascularization + heart failure)	Within 1 year after discharge	Events obtained from inpatient/outpatient records and telephone contact; PIV measured at multiple time points, with 12 h after PCI showing best AUC.
Bayramoğlu and Hidayet, 2023	Türkiye	Not reported (single outcome: no-reflow)	No-reflow / final TIMI flow <=2	-	-	-	-	Immediate post-PCI angiographic assessment	TIMI flow was assessed on post-procedure angiograms by two expert interventional cardiologists blinded to clinical data; no-reflow was defined as final TIMI flow <=2, conceptually overlapping with impaired coronary flow as defined by Şen et al. 2024.
Murat et al., 2023	Türkiye	Not reported (single outcome: all-cause mortality)	All-cause mortality	-	-	-	-	Mean follow-up 18.86 +/- 8.67 months; primary endpoint: 1-year all-cause mortality; secondary endpoints: in-hospital and	In-hospital, 1-month, and 1-year all-cause mortality described; no composite endpoint.

First author, year	Country	Composite outcome name as reported	Component 1	Component 2	Component 3	Component 4	Component 5+	Follow-up duration	Notes
								1-month all-cause mortality	
Zorlu et al., 2025	Türkiye	Not reported (single outcome: contrast-induced nephropathy)	Contrast-induced nephropathy	-	-	-	-	48-72 h after PCI	CIN defined as >=25% or >=0.5 mg/dL creatinine increase from baseline within 48-72 h; combined PIV + TyG index studied.

Composite cardiovascular outcomes were extracted as defined by each individual study, with components recorded verbatim as reported in the original publications. The composite outcome label used by each study (e.g., MACE, MACCE, NACE, or composite cardiovascular endpoint) is preserved as reported, without harmonisation across studies, in accordance with the study-defined composite outcome strategy specified in the registered review protocol (PROSPERO CRD420261378751). Studies that did not report a composite cardiovascular endpoint are indicated as such. Follow-up duration is presented as reported by the original study (median, mean, or maximum follow-up, or "in-hospital only" where applicable). This table supports the narrative synthesis of outcome heterogeneity discussed in the main text. Abbreviations: ACS, acute coronary syndrome; CA-AKI, contrast-associated acute kidney injury; HF, heart failure; IQR, interquartile range; MACCE, major adverse cardiac and cerebrovascular events; MACE, major adverse cardiovascular events; MI, myocardial infarction; NACE, net adverse clinical events; PCI, percutaneous coronary intervention; PIV, pan-immune-inflammation value; TLR, target lesion revascularization; TVR, target vessel revascularization.

Supplementary Table S4. Risk of bias assessment for individual studies using the Quality in Prognosis Studies (QUIPS) tool.

First author, year	(1) Study participation	(2) Study attrition	(3) Prognostic factor measurement	(4) Outcome measurement	(5) Study confounding	(6) Statistical analysis and reporting	Overall judgment	Supporting rationale
Çiçek et al., 2026	Moderate	Low	Low	Low	Moderate	Low	Moderate	Retrospective STEMI primary-PCI cohort with clear inclusion and a short creatinine follow-up window. Admission PIV and CA-AKI were measured using routine laboratory definitions. Confounding was partly addressed with multivariable modelling including age, Killip class and contrast volume, but residual renal and procedural confounding remains possible.
Keskin et al., 2026	Moderate	Low	Low	Low	High	High	High	Retrospective STEMI primary-PCI cohort with mortality ascertainment over 24 months. PIV was a candidate feature in machine-learning models rather than a prespecified standalone prognostic factor. No PIV-specific adjusted effect estimate or PIV-only discrimination result was identified, and the number of deaths was small relative to the modelling complexity. The high overall risk of bias rating reflects critical concerns in the study confounding and statistical analysis and reporting domains, which are the domains most directly relevant to the validity of PIV-outcome associations.
Qu et al., 2026	Moderate	Moderate	Low	Moderate	Moderate	Moderate	Moderate	Single-center retrospective STEMI-PCI cohort of 200 patients. PIV was measured from routine blood tests and MACE was assessed over 12 months, but the composite was described broadly as including but not limited to several events and adjudication was not reported. Adjustment was limited to selected clinical variables, and the high event proportion warrants reviewer checking.
Ma et al., 2025	Low	Low	Low	Moderate	Moderate	Moderate	Moderate	Prospective single-center cohort restricted to elderly AMI patients after PCI, with explicit eligibility criteria supporting low risk for study participation. PIV was measured from routine admission blood counts, and in-hospital heart failure was the outcome. The outcome was clinically relevant but adjudication details were limited, and adjustment may not fully capture infarct severity, renal function and procedural confounding.
Chen et al., 2025	Moderate	Moderate	Low	Moderate	Moderate	Moderate	Moderate	Retrospective cohort with a post-PCI MI subgroup followed for median 12 months. PIV measurement and MACE components were reported, but outcome adjudication and follow-up completeness were not clearly described. Multivariable logistic regression was performed, although adjustment was limited and residual confounding is likely.
Konte et al., 2025	Moderate	Low	Low	Low	Moderate	Moderate	Moderate	Retrospective ACS-PCI cohort with objective 48-72 h creatinine-based CI-AKI outcome. Baseline inflammatory indices including PIV were measured before intervention. PIV was significant in ROC analysis but not retained as an independent predictor in the multivariable model, so confounding and PIV-specific reporting need careful interpretation.
Xu et al., 2025	Low	Low	Low	Low	Moderate	Moderate	Moderate	Prospective STEMI-PCI study with missing data below 5% and blinded Clinical Events Committee (CEC) review of 6-month MACE, supporting low risk for study participation and outcome measurement. PIV was measured dynamically within the first 24 h after symptom onset, falling within the prespecified eligible measurement window of admission to early index hospitalization before outcome occurrence. Adjustment included selected biomarkers and GRACE score, but sample size and multiple dynamic predictors create modelling concerns.
Ting et al., 2025	Low	Low	Low	Low	Moderate	Low	Moderate	Retrospective STEMI primary-PCI cohort with mortality ascertained through the Taiwan National Cause of Death Registry and prespecified 1-year outcomes, supporting low risk for study participation and outcome measurement. PIV was calculated from initial complete blood count values. Multivariable Cox models and proportional hazards checks were reported, but variable selection and residual clinical confounding remain moderate concerns.
Liu et al., 2025	Moderate	Moderate	Low	Moderate	Moderate	Low	Moderate	Consecutive ACS-PCI cohort with long-term follow-up and explicit MACE components. Admission PIV indices were measured routinely, and Cox models adjusted for heart failure, cardiogenic shock, renal function, age and LVEF. Exclusion of patients lost to follow-up and lack of blinded endpoint adjudication support moderate rather than low risk.
Byoun et al., 2025	Moderate	Low	Low	Moderate	Moderate	Low	Moderate	Retrospective non-ST-elevation ACS PCI cohort with clear 1-year MACEs and individual components. PIV was measured from blood cell counts, and follow-up duration was reported. Outcome adjudication was not described, and confounding adjustment was present but incomplete for procedural and treatment-related factors.
Ömür et al., 2024	Moderate	Low	Low	Low	High	Moderate	High	Retrospective ACS cohort evaluating CIN and correlations between PIV, Mehran score and SYNTAX score. CIN was objectively defined within the expected post-contrast window, but the primary analyses relied mainly on ROC and correlation rather than a PIV-specific multivariable prognostic model. The lack of a PIV-specific multivariable model and reliance on ROC/correlation analyses raise critical concerns in the study confounding domain, which is one of the four critical domains determining the overall risk of bias rating; the overall judgment of high risk of bias reflects this critical-domain concern.
Genc et al., 2025	Low	Low	Low	Low	Moderate	Moderate	Moderate	Large STEMI pPCI cohort with derivation and validation cohorts and in-hospital all-cause mortality as an objective endpoint, supporting low risk for study participation. PIV and other indices were measured from admission laboratory tests. The main model centered on modified Naples Prognostic Score (mNPS), so PIV-specific independent prognostic interpretation is less direct despite generally adequate reporting.
Yang et al., 2024	Moderate	Low	Low	Moderate	Moderate	Low	Moderate	Retrospective STEMI-PCI cohort with in-hospital MACE definitions and 1-week follow-up. PIV was measured from routine blood counts and multivariable adjustment was reported in trend and subgroup analyses, but PIV-specific adjusted effect estimates and adjustment for full procedural confounders were not consistently presented. Outcome adjudication was not identified, and the short composite outcome mixes several clinically different events.
Kurtul and Gok, 2024	Moderate	Low	Low	Low	Moderate	Low	Moderate	Retrospective ACS-PCI cohort with objective creatinine-based PCAKI within 72 h. Preinterventional PIV was clearly measured and multivariable modelling adjusted for several clinical and procedural predictors. Single-center retrospective selection and residual confounding support moderate overall risk of bias.
Şen et al., 2024	Moderate	Low	Low	Low	Moderate	Low	Moderate	Retrospective STEMI pPCI cohort using angiographic ICF/no-reflow assessed by TIMI flow and myocardial blush criteria, checked by a blinded interventional cardiologist. PIV was measured before outcome assessment and multivariable models included clinical/procedural variables. Single-center design and residual confounding remain moderate concerns.
Liu et al., 2023	Moderate	Moderate	Low	Moderate	Moderate	Moderate	Moderate	Retrospective STEMI primary-PCI cohort with 1-year MACEs collected from records and telephone contact. Admission PIV was available and is the preferred measurement time point under the prespecified hierarchy, although the original publication highlighted better discrimination for 12 h post-PCI PIV. Loss-to-follow-up exclusion, non-adjudicated outcomes and PIV not being the final independent predictor support moderate overall risk of bias.
Bayramoğlu and Hidayet, 2023	Moderate	Low	Low	Low	Moderate	Low	Moderate	Retrospective STEMI-PCI cohort with no-reflow defined by final TIMI flow and assessed by two blinded interventional cardiologists. Admission PIV was evaluated with multivariable logistic regression and ROC analysis. Residual procedural confounding and single-center retrospective participation justify a moderate overall.
Murat et al., 2023	Moderate	Moderate	Low	Moderate	Moderate	Moderate	Moderate	Retrospective STEMI cohort with all-cause mortality endpoints at in-hospital, 1-month and 1-year time points and mean follow-up of about 19 months. PIV was measured at admission and multivariable survival modelling was reported. Outcome ascertainment methods (registry versus records) were not explicitly described, and incomplete adjustment for procedural and treatment factors supports moderate risk of bias.
Zorlu et al., 2025	Moderate	Low	Low	Low	High	Moderate	High	Retrospective STEMI-PCI cohort with routine pre-PCI PIV/TyG measurement and objective creatinine-based CIN within 48-72 h. The analysis focused on ROC performance and marker combinations, with no clear multivariable PIV-specific prognostic model identified. Excluding patients who died during PCI or within 72 h and limited confounding adjustment raise critical concerns in the study confounding domain. The overall high risk of bias judgment is anchored in this critical-domain finding.

Risk of bias was independently assessed by two reviewers using the Quality in Prognosis Studies (QUIPS) tool, which evaluates six domains specifically designed for prognostic factor research: study participation, study attrition, prognostic factor measurement, outcome measurement, study confounding, and statistical analysis and reporting. Each domain was rated as low, moderate, or high risk of bias based on the signaling questions and guidance provided in the QUIPS framework. Disagreements between reviewers were resolved through discussion and consensus, in accordance with the procedure specified in the registered review protocol (PROSPERO CRD420261378751). The overall risk of bias judgment was assigned with particular emphasis on the four domains most directly relevant to the validity of associations between the pan-immune-inflammation value and adverse cardiovascular outcomes, namely prognostic factor measurement, outcome measurement, study confounding, and statistical analysis and reporting. Supporting rationales summarise the key methodological features informing each overall judgment. Abbreviations: ACS, acute coronary syndrome; CI, confidence interval; eGFR, estimated glomerular filtration rate; LVEF, left ventricular ejection fraction; PCI, percutaneous coronary intervention; PIV, pan-immune-inflammation value; QUIPS, Quality in Prognosis Studies.

Supplementary Table S5. GRADE evidence profile for the primary and key secondary outcomes.

Outcome	No. of studies (No. of participants)	Pooled effect estimate (95% CI)	Risk of bias	Inconsistency	Indirectness	Imprecision	Publication bias	Large effect	Dose-response	Residual confounding	Starting level	Final certainty	Comments
Primary: Study-defined MACE	Adjusted association: k = 2 (n = 2,966; events = 169). Predictive performance: k = 5 (n = 2,103; events = 389).	Adjusted association: HR 1.65 (95% CI 1.20-2.27; I^2 = 0%). Predictive performance using prespecified PIV timing: AUC 0.729 (95% CI 0.578-0.814; I^2 = 83.0%).	Serious	Serious	Not serious	Not serious	Could not be formally assessed (k < 10)	No	Not applicable (downgrading present)	No	High	Low	This outcome includes two distinct evidence streams: prognostic association (adjusted HR) and discriminatory performance (AUC), each considered separately for GRADE domain judgments. The adjusted HR subset was small but directionally consistent, statistically significant, and showed no statistical heterogeneity. The AUC subset showed substantial between-study heterogeneity in discriminatory performance. REC00020 was recalibrated to admission PIV for the predictive-performance synthesis to align with the prespecified measurement hierarchy; the original 12 h post-PCI PIV estimate is retained as supportive but not primary. The final certainty rating reflects limitations in both evidence streams.The AUC evidence stream showed wider confidence intervals after Hartung–Knapp adjustment for k = 5 studies; the overall certainty rating was primarily informed by the adjusted hazard ratio evidence stream, which showed directionally consistent estimates with confidence intervals excluding the null.
All-cause mortality	Adjusted association: k=2 (n=2,295; events=97). AUC: one study reported PIV-specific in-hospital mortality AUCs (Genc et al. 2025; derivation 0.586; validation 0.710; overall 0.631; n=2,576) without 95% CIs, not eligible for AUC pooling.	Adjusted HR 3.51 (2.15–5.74; I^2=0%). PIV-specific discrimination reported as point estimates without 95% CIs in one study.	Not serious	Not serious for the compatible HR subset; not assessable for single-study AUC	Not serious	Serious	Could not be formally assessed (k < 10)	Not applicable (downgrading present)	No	No	High	Moderate	Both contributing studies were rated at moderate overall QUIPS risk with no high-risk studies, so risk of bias was not downgraded. Downgrading was concentrated on imprecision and the limited evidence base rather than RoB. The compatible high-vs-low PIV HR subset showed a large positive association with no statistical heterogeneity, but dose-response was clearly reported in only one mortality study and was not replicated across the mortality body of evidence.
Cardiovascular or cardiac mortality	Single component estimate from Byoun et al., 2025: k = 1 (n = 1,606; events = 24).	Adjusted association: HR 3.24 (95% CI 1.34-7.81).	Not serious	Not assessable (single study; not downgraded separately)	Not serious	Very serious	Could not be formally assessed (k < 10)	No (single study, 24 events; CI too wide to confidently confirm large magnitude)	No	No	High	Low	The contributing estimate came from Byoun et al., 2025, which reported 1-year cardiac death as an individual component of MACE in NSTE-ACS patients. Liu et al., 2025 reported cardiogenic mortality only as part of the composite MACE endpoint and did not provide a separate PIV-specific component estimate, so it was not included in this row. The contributing study was rated at moderate overall QUIPS risk, so the RoB domain was not downgraded separately. Certainty was downgraded primarily for very serious imprecision because the evidence relied on a single component-level estimate with only 24 cardiac-death events and a wide CI. The GRADE framework is therefore applied with caution for this single-study evidence body.
No-reflow or slow-flow	Predictive performance: k = 2 (n = 1,899; events = 262). Association estimates were not pooled because one study reported high-vs-low PIV and the other reported continuous PIV.	Predictive performance: AUC 0.828 (95% CI 0.808-0.846; I^2 = 0.0%).	Not serious	Not serious	Not serious	Serious	Could not be formally assessed (k < 10)	No	No	No	High	Moderate	Both contributing studies were rated at moderate overall QUIPS risk with no high-risk studies, and their AUC estimates were highly consistent. Downgrading was concentrated on imprecision because the evidence base was limited to two retrospective STEMI pPCI studies. Association estimates were retained narratively because exposure scaling was not comparable across studies.
Contrast-associated AKI / contrast-induced nephropathy	Predictive performance: k = 5 (n = 7,541; events = 1,996). Exploratory high-vs-low adjusted OR subset: k = 2 (n = 3,164; events = 617).	Predictive performance: AUC 0.771 (95% CI 0.617-0.875; I^2 = 95.1%). Excluding high-risk studies: AUC 0.790 (95% CI 0.656-0.881; I^2 = 93.6%). Exploratory adjusted OR 4.91 (95% CI 0.87-27.89; I^2 = 90.3%).	Serious	Serious	Not serious	Serious	Could not be formally assessed (k < 10)	No	No	No	High	Very low	Risk of bias was downgraded because 2 of 5 studies (Ömür 2024 and Zorlu 2025) were rated at high overall QUIPS risk, primarily due to insufficient PIV-specific multivariable adjustment. AUC results consistently favored PIV above chance discrimination, but between-study heterogeneity was very high. Imprecision was rated Serious overall, driven primarily by the very wide CI of the exploratory adjusted OR subset; the main AUC pooled estimate alone would not warrant downgrading on imprecision. With three serious downgrading domains, the final certainty is very low.

Certainty of evidence was assessed using the Grading of Recommendations Assessment, Development and Evaluation (GRADE) approach, adapted for prognostic factor systematic reviews. For each outcome, the body of evidence was evaluated across five domains for downgrading—risk of bias, inconsistency, indirectness, imprecision, and publication bias—and three domains for upgrading—large magnitude of effect, dose-response gradient, and plausible residual confounding that would reduce rather than inflate the observed association. The starting certainty for observational evidence on prognostic factor associations was set at high, consistent with current guidance for prognostic factor systematic reviews. Each outcome was assigned an overall certainty rating of high, moderate, low, or very low. Disagreements between reviewers were resolved through discussion and consensus, in accordance with the procedure specified in the registered review protocol (PROSPERO CRD420261378751).

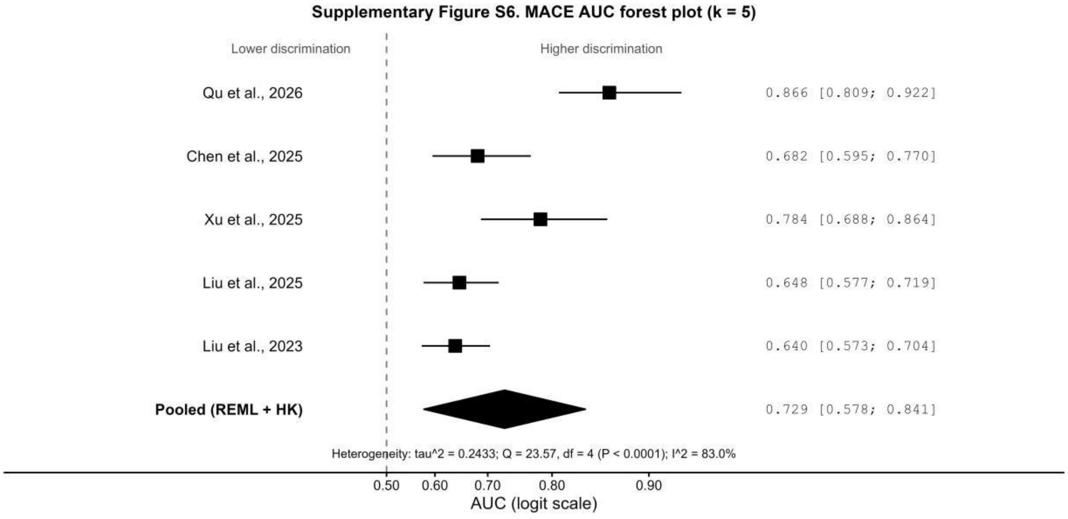

Supplementary Figure S6. Forest plot of the area under the receiver operating characteristic curve (AUC) for the discrimination of study-defined major adverse cardiovascular events (MACE) by pan-immune-inflammation value (PIV) in patients with acute coronary syndrome undergoing percutaneous coronary intervention. Estimates were pooled using random-effects meta-analysis with restricted maximum likelihood (REML) estimation of the between-study variance, on the logit-transformed AUC scale, with Hartung–Knapp adjustment for the confidence interval (k = 5). The x-axis is displayed on the logit scale with reference labels showing the corresponding AUC values; the reference line at logit(AUC) = 0 corresponds to an AUC of 0.50, indicating no discrimination. Squares represent study-level AUCs with 95% confidence intervals; the diamond represents the pooled estimate. AUC, area under the receiver operating characteristic curve; CI, confidence interval; PIV, pan-immune-inflammation value.

Supplementary Table S7. Sensitivity analyses: leave-one-out and fixed-effect versus random-effects re-analysis.

Part 1. Leave-one-out re-pooling for outcomes with five contributing studies.

Outcome	Excluded study	k after exclusion	Pooled estimate (95% CI)	I^2 (%)
**MACE AUC**	None (full pool)	5	0.729 (0.578 to 0.841)	83.0
	Qu et al., 2026	4	0.677 (0.570 to 0.769)	49.1
	Chen et al., 2025	4	0.742 (0.520 to 0.884)	87.2
	Xu et al., 2025	4	0.717 (0.494 to 0.867)	85.2
	Liu et al., 2025	4	0.750 (0.544 to 0.883)	85.7
	Liu et al., 2023	4	0.752 (0.551 to 0.882)	84.3
**CA-AKI AUC**	None (full pool)	5	0.771 (0.617 to 0.875)	95.1
	Çiçek et al., 2026	4	0.776 (0.538 to 0.911)	96.0
	Konte et al., 2025	4	0.790 (0.589 to 0.908)	96.2
	Ömür et al., 2024	4	0.760 (0.523 to 0.902)	95.4
	Kurtul and Gok, 2024	4	0.731 (0.604 to 0.828)	93.0
	Zorlu et al., 2025	4	0.795 (0.604 to 0.908)	91.4

Part 2. Fixed-effect versus random-effects re-analysis.

Outcome	k	Random-effects estimate (95% CI)	Fixed-effect estimate (95% CI)	Comment
**MACE adjusted HR**	2	1.65 (1.20 to 2.27)	1.65 (1.20 to 2.27)	Identical (I^2 = 0%)
**MACE AUC**	5	0.729 (0.578 to 0.841)	0.695 (0.659 to 0.730)	Fixed-effect CI substantially narrower; I^2 = 83.0%
**All-cause mortality HR**	2	3.51 (2.15 to 5.74)	3.51 (2.15 to 5.74)	Identical (I^2 = 0%)

Outcome	k	Random-effects estimate (95% CI)	Fixed-effect estimate (95% CI)	Comment
**No-reflow AUC**	2	0.828 (0.808 to 0.846)	0.828 (0.808 to 0.846)	Identical ($I^2 = 0\%$)
**CA-AKI AUC**	5	0.771 (0.617 to 0.875)	0.730 (0.714 to 0.745)	Fixed-effect CI substantially narrower; $I^2 = 95.1\%$
**CA-AKI adjusted OR** (exploratory)	2	4.91 (0.87 to 27.89)	3.21 (2.03 to 5.08)	Fixed-effect CI excludes the null but does not adequately represent uncertainty; $I^2 = 90.3\%$

Sensitivity analyses were performed by two reviewers for outcomes with five contributing studies (leave-one-out re-pooling for MACE AUC and CA-AKI AUC) and across all six pooled analyses (fixed-effect versus random-effects re-analysis). All analyses were performed in R using the meta package, in accordance with the methods specified in the registered review protocol (PROSPERO CRD420261378751). Random-effects models used the restricted maximum likelihood (REML) estimator of the between-study variance, with Hartung–Knapp adjustment applied for the two analyses with five contributing studies and not applied for the analyses with two contributing studies, as prespecified. Areas under the receiver operating characteristic curve were pooled on the logit-transformed scale; hazard and odds ratios were pooled on the natural logarithmic scale. Confidence intervals on transformed scales were back-transformed for presentation.
